# Supplementary material for: Increases in cyclin A/Cdk activity and in PP2A-B55 inhibition by FAM122A are key mitosis-inducing events
Source: EMBO J. 2024 Feb 20;43(6):993–1014. doi: 10.1038/s44318-024-00054-z (PMC10943098; doi:10.1038/s44318-024-00054-z)
Supplement: Supplementary file 1 — Appendix [file 44318_2024_54_MOESM1_ESM.pdf]

**APPENDIX MATERIALS** **Pages**

Contents


| | |
|---|---|
| **Contents** | **1** |
| **Appendix Figures S1-S6** | **2-10** |
| **Appendix Table S1-2** | **11-14** |
| **Appendix references** | **15** |

# Appendix Figure S1

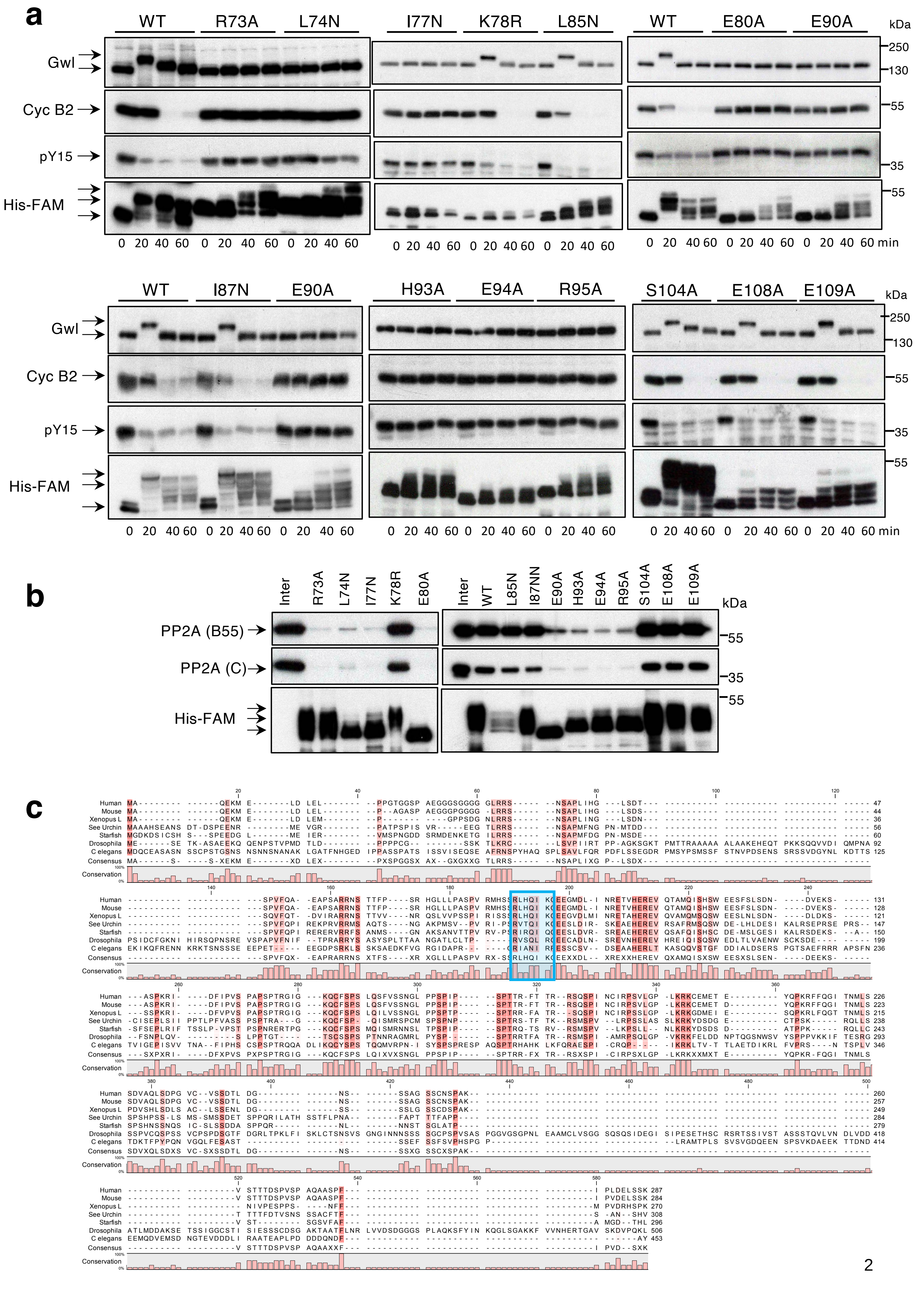

**Appendix Figure S1, related to Fig.3**

**Identification of the residues of αH1 and αH2 required to PP2A inhibitory activity of Xe FAM122A. (a)** The capacity of the indicated mutants of Xe FAM122A to promote mitotic entry when supplemented to interphase extracts is shown. **(b)** B55/C binding to the single mutants of the αH1 and αH2 is measured by His-pull down. **(c)** FAM122A sequence alignment on different species. Putative SLiM motif is highlighted in blue.

# Appendix Figure S2

a

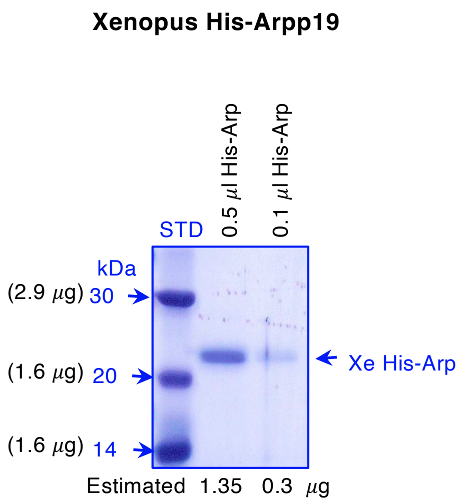


b

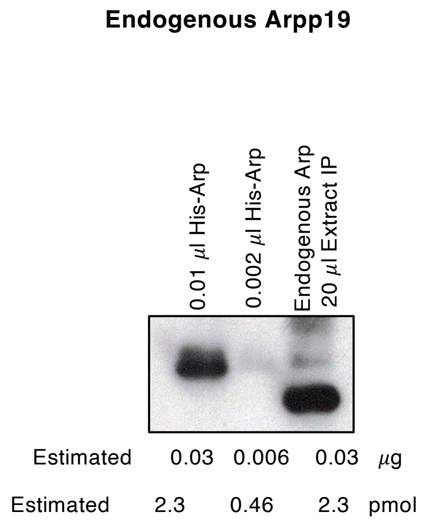


**Concentration in the extracts:** 2.3 pmol x 1/20= 0.100 pmol/µl

**Molarity in the extracts:** 0.1 µ M

c

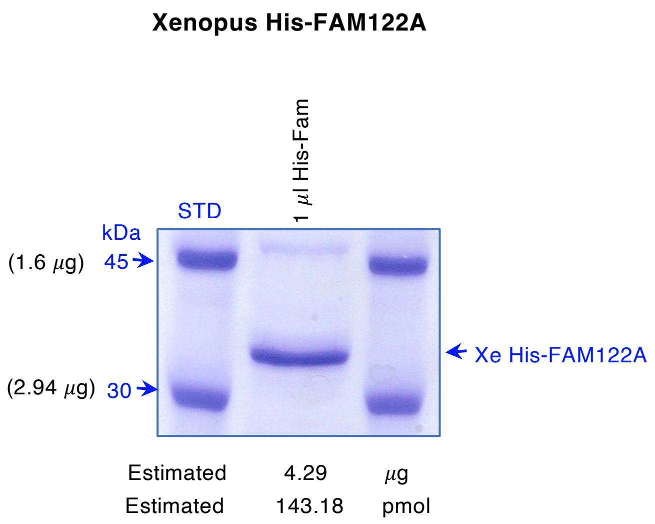


**Concentration:** 143.18 pmol /µl

**Volume used :** 3 µl

**Total amount used :** 429.54 pmol

**Molarity when added to 20 µl of extracts**: 21.5 µ M

**Appendix Figure S2, related to Fig.5**

**Estimation of final concentration in the extracts of endogenous Arpp19 and of the His-FAM122A used to induce mitotic entry. (a)** The indicated volumes of the His-Arpp19 purified protein were used for PAGE-SDS and Coomassie blue staining. Intensity bands corresponding to His-Arpp19 were compared to standard markers and the protein amount quantified using ImageJ and estimated. **(b)** 20 µl of extracts were immunoprecipitated using anti-Arpp19 antibodies and submitted to western blot and the Arpp19 signal was compared with the one obtained for 0,03 and 0,006 µg of recombinant Xe-His-Arpp19. **(c)** Xenopus His-FAM122A concentration was estimated as for (a) and the final molarity corresponding to 1µl of His-FAM122A in 20 µl of extracts used for the experiments calculated.

# Appendix Figure S3

## a Inhibition of PP2A-B55 by FAM122A

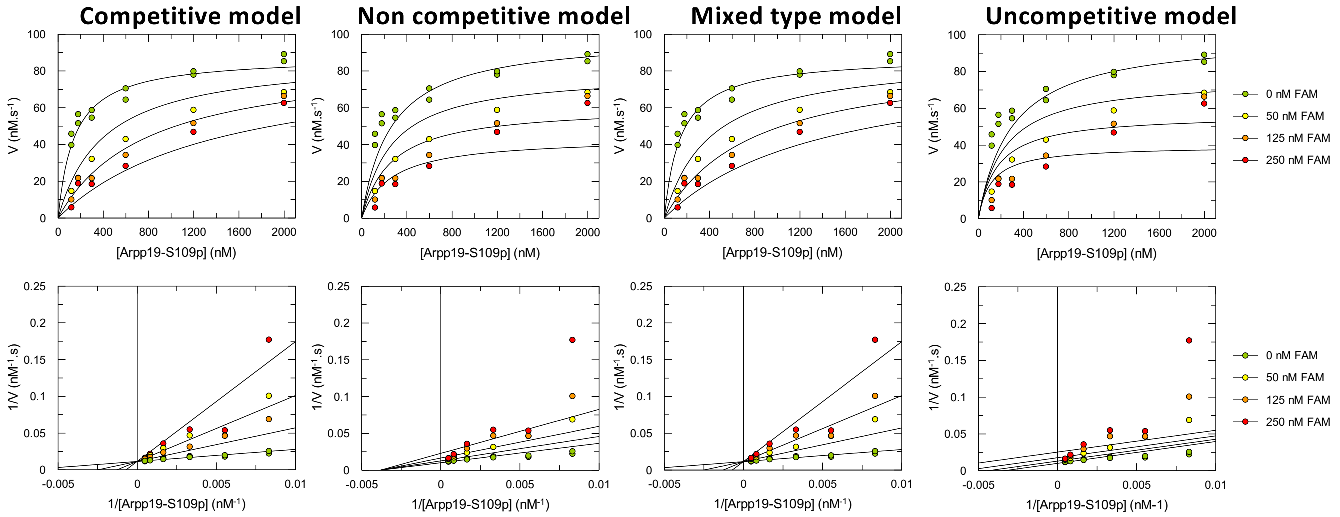


**Competitive model** — Reduced Chi²: 30.4

| Parameter | Value | Std. Error |
|---|---|---|
| Vmax | 87.9 | 3.2 |
| Km | 144.6 | 21.9 |
| Ki | 28.1 | 4.6 |

**Non competitive model** — Reduced Chi²: 91.4

| Parameter | Value | Std. Error |
|---|---|---|
| Vmax | 99.2 | 6.6 |
| Km | 262.3 | 51.7 |
| Ki | 198.0 | 35.7 |

**Mixed type model** — Reduced Chi²: 31.6

| Parameter | Value | Std. Error |
|---|---|---|
| Vmax | 87.9 | 3.3 |
| Km | 144.6 | 22.4 |
| Ki | 28.1 | 4.7 |
| Ki' value | 5.0e+025 | 0.0 |

**Uncompetitive model** — Reduced Chi²: 130.9

| Parameter | Value | Std. Error |
|---|---|---|
| Vmax | 99.8 | 9.0 |
| Km | 296.6 | 76.2 |
| Ki | 163.7 | 43.0 |

## b Inhibition of PP2A-B55 by Thio-S71-Arpp19

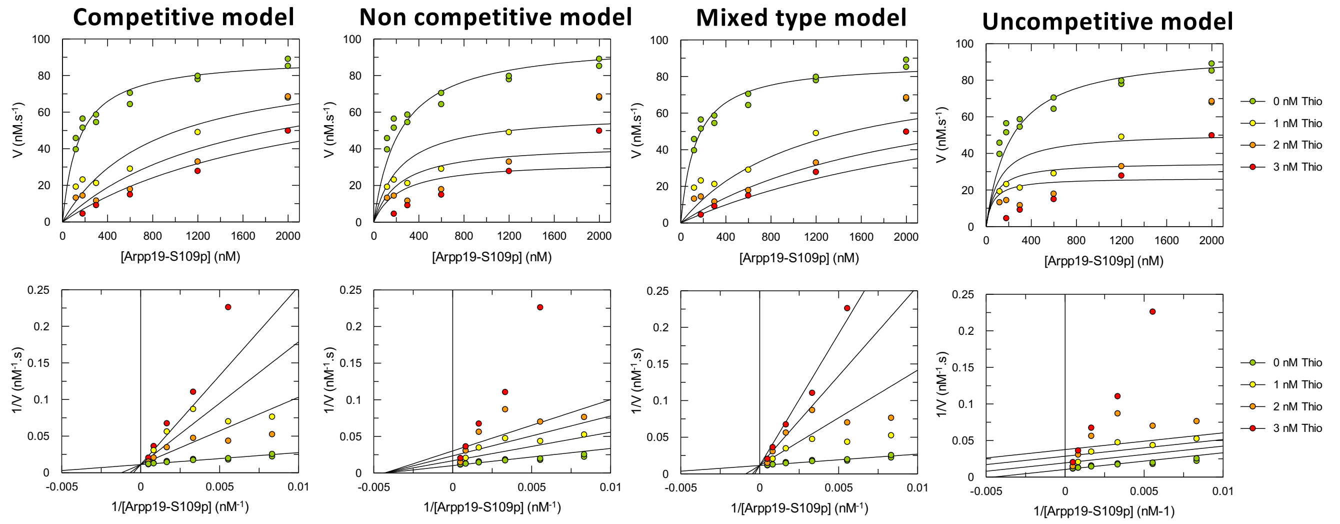


**Competitive model** — Reduced Chi²: 39.37

| Parameter | Value | Std. Error |
|---|---|---|
| Vmax | 90.19 | 3.99 |
| Km | 147.89 | 26.05 |
| Ki | 0.22 | 0.04 |

**Non competitive model** — Reduced Chi²: 117.08

| Parameter | Value | Std. Error |
|---|---|---|
| Vmax | 98.91 | 7.67 |
| Km | 232.25 | 56.54 |
| Ki | 1.52 | 0.27 |

**Mixed type model** — Reduced Chi²: 67.91

| Parameter | Value | Std. Error |
|---|---|---|
| Vmax | 88.11 | 5.31 |
| Km | 136.69 | 33.66 |
| Ki | 0.14 | 0.05 |
| Ki' value | 1.0e+011 | 6.0e+01[illegible] |

**Uncompetitive model** — Reduced Chi²: 164.92

| Parameter | Value | Std. Error |
|---|---|---|
| Vmax | 96.35 | 9.55 |
| Km | 220.44 | 71.54 |
| Ki | 1.14 | 0.28 |

**Appendix Figure S3, related to Fig 6.**

**Determination of the mode of inhibition of PP2A-B55 by FAM122A and p-S71-Arpp19. (a)** Inhibition of PP2A-B55 by FAM122A. The inhibition of PP2A-B55 by FAM122A was fitted using 4 different inhibition models: competitive of p-S113-Arpp19, non-competitive, mixed or uncompetitive. Lineweaver-Burk representations, reduced Chi$^2$ values and the fitting results are shown below each panel. **(b)** Inhibition of PP2A-B55 by Thio-S71-Arpp19 was fitted as for (a).

# Appendix Figure S4

## a Inhibition of PP2A-B55 byFAM122A

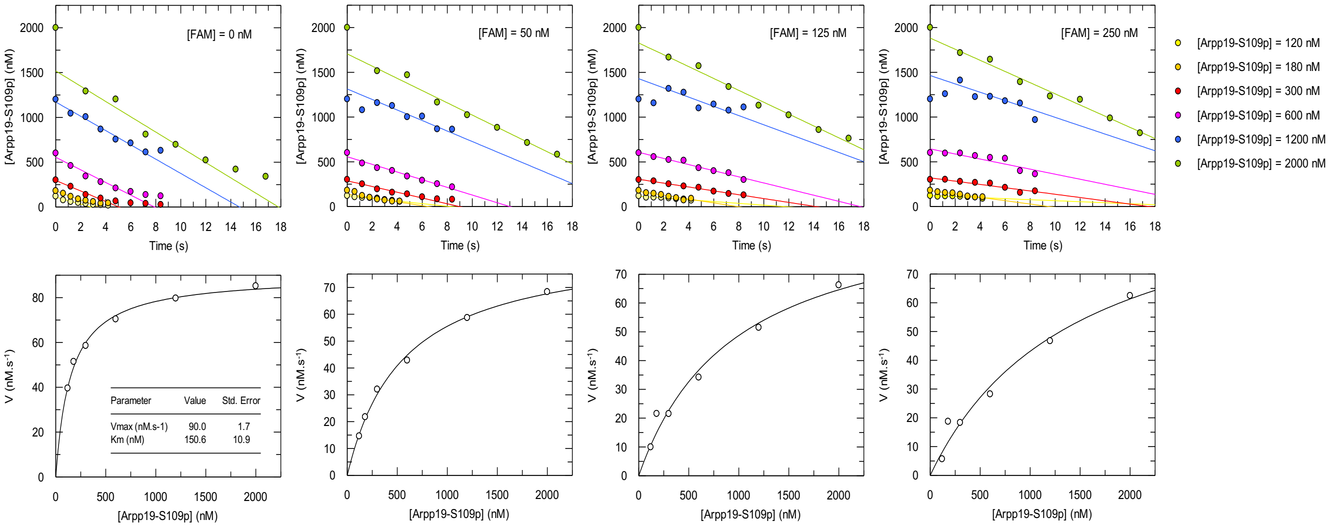


Reduced Chi²: 30.4

| Parameter | Value | Std. Error |
|---|---|---|
| Vmax | 87.9 | 3.2 |
| Km | 144.6 | 21.9 |
| Ki | 28.1 | 4.6 |

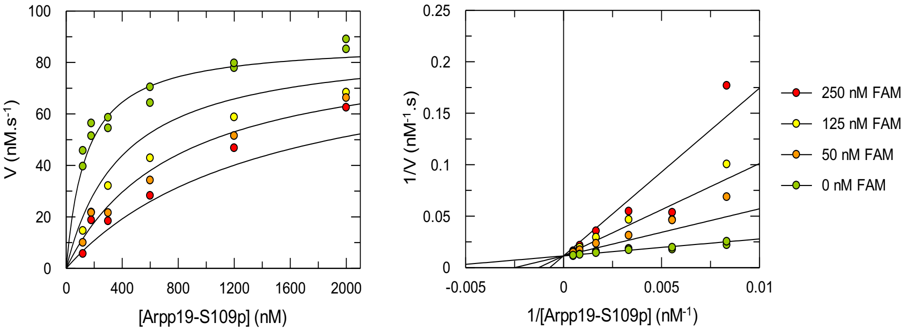


## b Inhibition of PP2A-B55 by Thio-S71-Arpp19

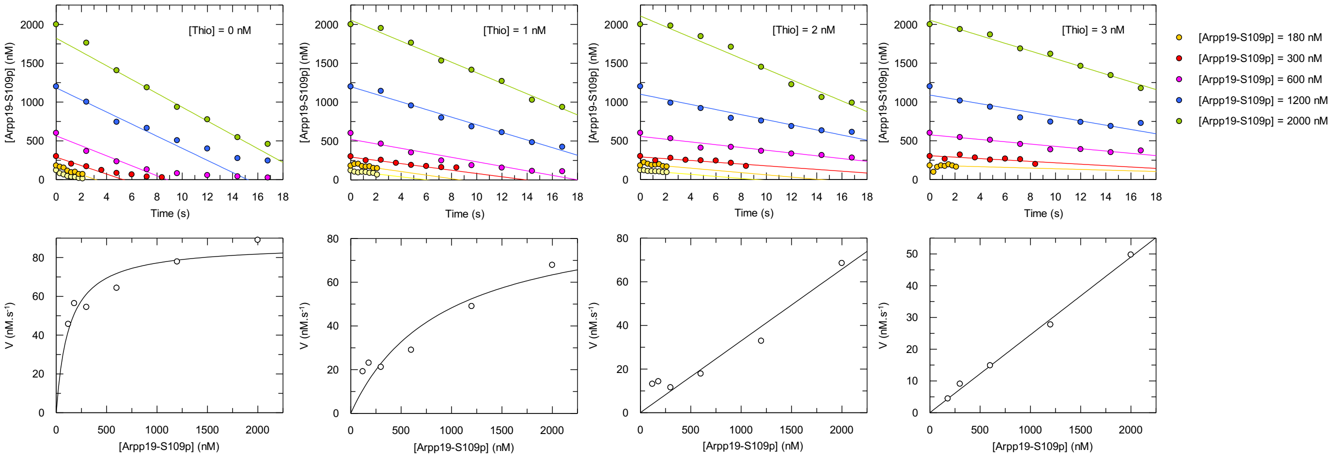


Reduced Chi²: 39.37

| Parameter | Value | Std. Error |
|---|---|---|
| Vmax | 90.19 | 3.99 |
| Km | 147.89 | 26.05 |
| Ki | 0.22 | 0.04 |

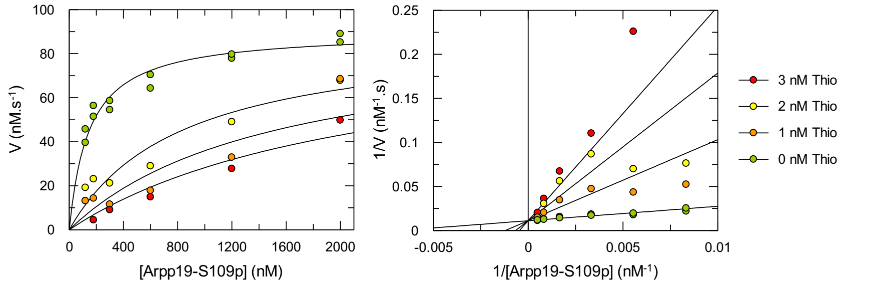

**Appendix Figure S4, related to Fig6.**

**Competitive inhibition of p-S113-Arpp19 by Fam122A and p-S71-Arpp19. (a)** Top panels show the time courses for the dephosphorylation of p-S113-Arpp19 (concentrations indicated in the legend) by PP2A-B55 and with different concentrations of FAM122A (indicated on the graphs). Below are shown the hyperbolic fittings of the rate constants determined for each series individually. The lower panels show the global fitting of the 4 series according to a competitive model and the corresponding Lineweaver-Burk representation. **(b)** As for (a) except that the thio-S71-Arpp19 inhibitor was used.

# Appendix Figure S5

**a**

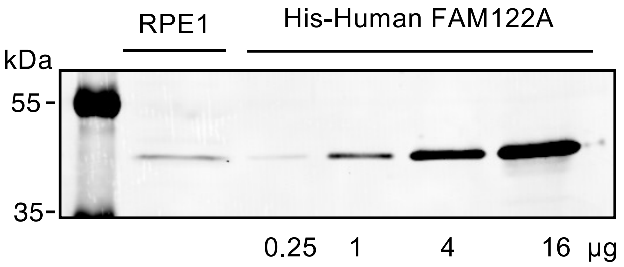


**Loading:** volume corresponding to 107,000 RPE1 cells

**Volume of RPE1 cells**: 2 pl

| | Exp. 1 | Exp. 2 | Exp. 3 | Mean | SD |
|---|---|---|---|---|---|
| Estimated FAM122A Amount (ng) | 0.37 | 0.44 | 0.34 | **0.38** | **0.05** |
| FAM122A Concentration (nM) | 57.6 | 68.5 | 52.9 | **59.6** | **6.37** |

**Appendix Figure S5, related to Fig.5.**

**Estimation of the final concentration of endogenous FAM122A in RPE1 cells. (a)** A volume corresponding to 107,000 RPE1 cells was loaded, together with 0.25, 1, 4 and 16 µg of His-Human FAM122A in a SDS-PAGE gel to perform western blot. Western blot signals were measured using ImageJ software and used to estimate endogenous FAM122A amount. FAM122A concentration was calculated by using a volume of 2 pl per RPE1 cell previously calculated in Cadart et *al.* (Cadart *et al*, 2018). Values of three different experiments were used to calculate a mean value ± standard deviation.

# Appendix Figure S6

**a**

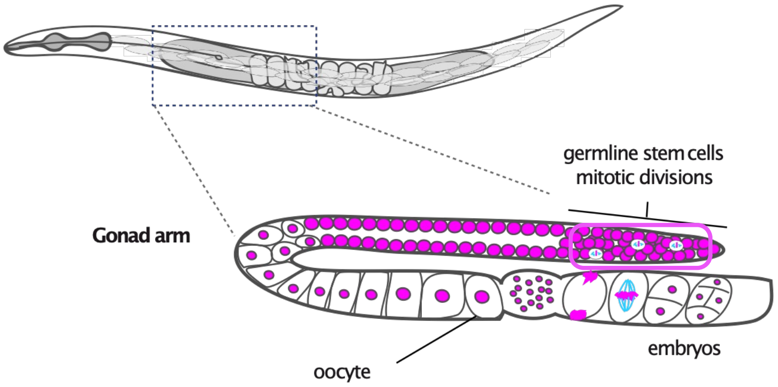


**b**

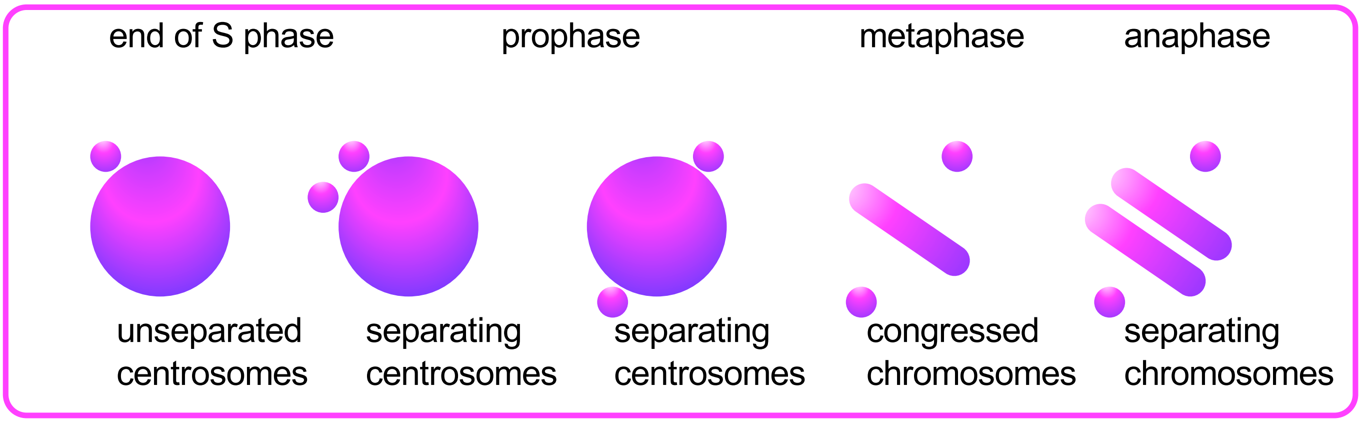


**c**

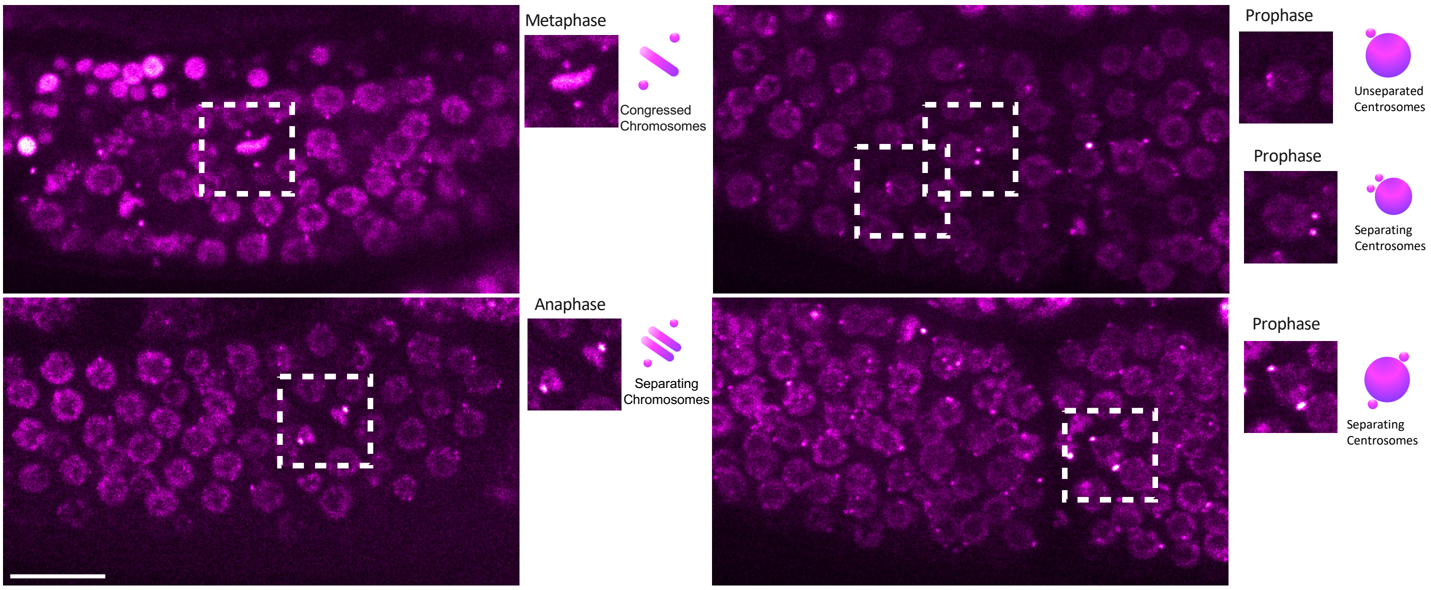

**Appendix Figure S6, related to Figure 7-8.**

**CeFAM122A depletion impairs mitotic entry of germ line stem cells in *C. elegans* nematodes.**

**(a)** Schematic representation of *C. elegans* nematode reproductive system. Germ line stem cells are located in the most distal part of each gonad arm. Chromatin is represented in magenta, microtubules in cyan **(b)** Schematic representation of the mitotic features observed and quantified based on chromatin and gamma-tubulin markers in Figure 8. **(c)** Representative images of the germ line stem cells imaged in TH32 strain (GFP-gamma-tubulin and GFP-histone) in Ce FAM122A depleted worms (right panel) or control worms (left panel). White dashed squares represent representative mitotic phases highlighted in zoomed square images next to each panel.

## Appendix Table S1

Antibodies, Bacterial Strains, Chemicals and Recombinant Proteins used in this study.

| REAGENT or RESOURCE | SOURCE | IDENTIFIER |
|---|---|---|
| **Antibodies** | | |
| Rabbit Polyclonal anti-Human Greatwall | Burgess et al. 2010 | N/A |
| Mouse Monoclonal anti-Histidine | Roche | Cat#11922416001 |
| Rabbit Polyclonal anti-Xenopus Cdc27 | Lorca et al. 2010 | N/A |
| Rabbit Polyclonal anti-Xenopus Cdc25 | Lorca et al. 2010 | N/A |
| Rabbit Polyclonal anti-Xenopus Myt1 | Vigneron et al 2009 | N/A |
| Rabbit Polyclonal anti-Xenopus cyclin A1 | Lorca et al. 2010 | N/A |
| Rabbit Polyclonal Phospo-S62/67 Arpp19 | Cell Signaling Technology | Cat#5240S |
| Rabbit Polyclonal Phospho-Cdc2 (Tyr-15) | Cell Signaling Technology | Cat#9111 |
| Rabbit Polyclonal anti-Xenopus Wee1 | Vigneron et al 2009 | N/A |
| Rabbit Polyclonal anti-Xenopus Cyclin B2 | Abrieu et al. 1997 | N/A |
| Rabbit Polyclonal anti-Xenopus Arpp19 | Ma et al. 2016 | N/A |
| Rabbit Polyclonal anti-PP2A/B55δ | Cell Signaling | Cat#2290 |
| Mouse Monoclonal anti-PP2A (C) subunit, alpha isoform | Merck Millipore | Cat#05-421 |
| Rat Polyclonal PP2A (A) subunit | Cell Signaling | Cat#2260 |
| Rabbit Polyclonal anti-PRC1 | Santa Cruz | Cat# 376982 |
| Goat Polyclonal anti-phosphorylated PRC1 (T481) | Santa Cruz | Cat#11768 |
| Anti-Cter Cdk1 | Labbé et al. 2021 | N/A |
| Rabbit Polyclonal anti-phosphorylated Arpp19 (S113) | Labbé et al. 2021 | N/A |
| Goat anti-rat IgG-HRP | Santa Cruz | Cat#2006 |
| HRP conjugated anti-Rabbit secondary antibodies | Cell Signalling Technology | Cat#7074 |
| Donkey anti-goat IgG-HRP | Santa Cruz | Cat#2020 |
| HRP conjugated anti-Mouse secondary antibodies | BioRad | Cat#1172-1011 |
| **Bacterial and Virus Strains** | | |

| | | |
|---|---|---|
| BL21DE3 Competent E.Coli | New England Biolabs | Cat#C2527H |
| DH5α E. Coli | New England Biolabs | Cat#C2987I |
| HT115(DE3) E. coli | Caenorhabditis Genetics Center (CGC) | http://www.cgc.cbs.umn.edu/strain.php?id=8854 |
| **Chemicals, Peptides, and Recombinant Proteins** | | |
| [gamma-P33] ATP | HARTMANN ANALYTIC | Cat# SRF-301 |
| Pfu ultra II fusion DNA polymerase | Agilent | Cat#600670 |
| $ATP^{\gamma S}$ | Sigma | Cat#A1388 |
| TALON Superflow Metal Affinity Resin | Takara | Cat#635506 |
| PVDF transfer membrane | Millipore | Cat#88518 |
| Protan Nitrocellulose membrane | Amersham | Cat#GE10600016 |
| Dynabeads protein G | Life Technologies | Cat#10004D |
| BSA | Sigma | Cat#A7906 |
| His-Pure™NiNTA magnetic beads | Life Technologies | Cat#88832 |
| Recombinant GST-Human Greatwall K72M mutant | Vigneron et al. 2011 | N/A |
| **Experimental Models: Organisms/Strains** | | |
| Xenopus Laevis | Centre de Ressources Biologiques Xenopes-Rennes | http://www.celphedia.eu/en/centers/crb |

## Appendix Table S2

Oligonucleotides, Recombinant DNA, Software and Sequence of Xe FAM122A S/T-to A mutant.

| **Oligonucleotides** | | |
|---|---|---|
| Forward and reverse primers for Xe FAM122A Δ(110-270):<br>5’ AGCCAGTCCTGGGAGGAATAACTAAATCTGAGTGACAATG 3’<br>5’ CATTGTCACTCAGATTTAGTTATTCCTCCCAGGACTGGCT 3’ | Eurogentec | This study |
| Forward and reverse primers for Xe FAM122A R73A:<br>5'ATACGTATCTCAAGCAGCGCTCTTCACCAGATTAAACAG 3'<br>5' CTGTTTAATCTGGTGAAGAGCGCTGCTTGAGATACGTAT 3' | Eurogentec | This study |
| Forward and reverse primers for Xe FAM122A L74N:<br>5’ CGTATCTCAAGCAGCCGTAATCACCAGATTAAACAGGA 3’<br>5’ TCCTGTTTAATCTGGTGATTACGGCTGCTTGAGATACG 3’ | Eurogentec | This study |
| Forward and reverse primers for Xe FAM122A I77N:<br>5’ AGCAGCCGTCTTCACCAGAATAAACAGGAAGAGGGAGTG 3’<br>5’ CACTCCCTCTTCCTGTTTATTCTGGTGAAGACGGCTGCT 3’ | Eurogentec | This study |
| Forward and reverse primers for Xe FAM122A E80A:<br>5’ CTTCACCAGATTAAACAGGCAGAGGGAGTGGACCTTATG 3’<br>5’ CATAAGGTCCACTCCCTCTGCCTGTTTAATCTGGTGAAG 3’ | Eurogentec | This study |
| Forward and reverse primers for Xe FAM122A L85N:<br>5’ CAGGAAGAGGGAGTGGACAATATGATTAACAGAGAGACG 3’<br>5’ CGTCTCTCTGTTAATCATATTGTCCACTCCCTCTTCCTG 3’ | Eurogentec | This study |
| Forward and reverse primers for Xe FAM122A I87N:<br>5’ GAGGGAGTGGACCTTATGAATAACAGAGAGACCGCTCATG 3’<br>5' CATGAGCGGTCTCTCTGTTATTCATAAGGTCCACTCCCTC 3’ | Eurogentec | This study |
| Forward and reverse primers for Xe FAM122A E90A:<br>5’ GACCTTATGATTAACAGAGCGACCGCTCATGAAAGGGAAG 3’<br>5' CTTCCCTTTCATGAGCGGTCGCTCTGTTAATCATAAGGTC 3’ | Eurogentec | This study |
| Forward and reverse primers for Xe FAM122A H93A:<br>5’ ATTAACAGAGAGACCGCTGCTGAAAGGGAAGTGCAAGTGG 3’<br>5’ CCACTTGCACTTCCCTTTCAGCAGCGGTCTCTCTGTTAAT 3’ | Eurogentec | This study |
| Forward and reverse primers for Xe FAM122A E94A:<br>5’ CAGAGAGACCGCTCATGCAAGGGAAGTGCAAGTGGCAA 3’<br>5’ TTGCCACTTGCACTTCCCTTGCATGAGCGGTCTCTCTG 3’ | Eurogentec | This study |
| Forward and reverse primers for Xe FAM122A R95A:<br>5’ CAGAGAGACCGCTCATGAAGCGGAAGTGCAAGTGGCAATG 3’<br>5’ CATTGCCACTTGCACTTCCGCTTCATGAGCGGTCTCTCTG 3’ | Eurogentec | This study |
| Forward and reverse primers for Xe FAM122A S104A:<br>5’ CAAGTGGCAATGCAAATGGCCCAGTCCTGGGAGGAAAGCC 3’<br>5’ GGCTTTCCTCCCAGGACTGGGCCATTTGCATTGCCACTTG 3’ | Eurogentec | This study |
| Forward and reverse primers for Xe FAM122A E108A:<br>5’CAAATGAGCCAGTCCTGGGCGGAAAGCCTAAATCTGAGTG 3’<br>5’ CACTCAGATTTAGGCTTTCCGCCCAGGACTGGCTCATTTG 3’ | Eurogentec | This study |
| Forward and reverse primers for Xe FAM122A E109A:<br>5’ ATGAGCCAGTCCTGGGAGGCAAGCCTAAATCTGAGTGAC3’<br>5’ GTCACTCAGATTTAGGCTTGCCTCCCAGGACTGGCTCAT3’ | Eurogentec | This study |
| **Recombinant DNA** | | |
| pET7F1-human Cyclin A | Generous gift of G Draetta. Lorca et al. 1992 | N/A |

| | | |
|---|---|---|
| pET15-6His-Xenopus Arpp19 | Ma et al .2016 | N/A |
| pFastBac-GST-hGwlK72M | Vigneron et al. 2011 | N/A |
| pET15b-human PKA | Labbé et al. 2021 | N/A |
| pET14b-Xenopus FAM122A | This study | N/A |
| pET15b-human PRC1 | Labbé et al. 2021 | N/A |
| pET14b-*C elegans* FAM122A | This study | N/A |
| pET14b-human FAM122A | This study | N/A |
| pET14b-Xenopus S/T-A FAM122A mutant | This study | N/A |
| **Software and Algorithms** | | |
| Adobe Photoshop | Microsoft | Version 12.0x64 |
| PowerPoint | Microsoft | Version 14.7.7 |
| ImageJ | NIH | 1.50i |

## Sequence of Xe FAM122A S/T-to-A mutant

gca cag gag aaa atg gag ctg gac ctc gaa atg ccg ggg ccc ccg **gct** gat ggg aat ctg agg agg **gct** aat **gcc** gcc cct ctc atc aat ggc ctc **gct** gac aat **gca** cag gtg ttt cag **gca** gat gtt ata aga gcg cgt cgg aac **gct gcc gct** gta gtg aat cgt cag **gct** ctg gtt gtg cca **gct gct** cca ata cgt atc **gca gcc gcc cgt ctt cac cag att aaa cag gaa gag gga gtg gac ctt atg att aac aga gag gcc gct cat gaa agg gaa gtg caa gtg gca atg caa atg gcc cag gcc tgg gag gaa gcc cta aat ctg gct gac aat gat ctc gat aaa gca gct gct cca aag aga att gat ttt gtt cca gtg gct cca gct cct gct ccc gca aga gga att gga aag caa tgc ttt gca cca gca tta caa ata ctt gtc gcc gcc aat gga ttg cca ccc gcc cct gcc cct gca ccc gca agg cgg ttt gca gcc agg cgg gct caa gct cca ata aac tgc att cga ccc gcc gca ctt gga cct ctt aaa cgg aaa gga gac atg gag att gaa gct cag cca aaa cga ctt ttc caa gga gct gca aac atg ctg gct cca gat gtc gct cac tta gca gat cta gcc gct tgt ctt gcc gcg gaa aat ctg gat gga gcc gcc gcc gcc ctt ggc gcg gcc tgc gac gct cct gct aaa aac att gtt ccc gaa gct cct cct gcc aac ttc ttt atg cca gtg gac aga cac gct cct aag tga**

## CONTACT FOR REAGENT AND RESOURCE SHARING

Further information and requests for resources and reagents should be directed to and will be fulfilled by the Lead Contact Thierry Lorca (thierry.lorca@crbm.cnrs.fr)

**Appendix References**

Abrieu A, Fisher D, Simon MN, Doree M & Picard A (1997) MAPK inactivation is required for the G2 to M-phase transition of the first mitotic cell cycle. *EMBO J* 16: 6407–13

Burgess A, Vigneron S, Brioudes E, Labbe JC, Lorca T & Castro A (2010) Loss of human Greatwall results in G2 arrest and multiple mitotic defects due to deregulation of the cyclin B-Cdc2/PP2A balance. *Proceedings of the National Academy of Sciences of the United States of America* 107: 12564–9

Cadart C, Monnier S, Grilli J, Sáez PJ, Srivastava N, Attia R, Terriac E, Baum B, Cosentino-Lagomarsino M & Piel M (2018) Size control in mammalian cells involves modulation of both growth rate and cell cycle duration. *Nat Commun* 9: 3275

Labbé JC, Vigneron S, Méchali F, Robert P, Roque S, Genoud C, Goguet-Rubio P, Barthe P, Labesse G, Cohen-Gonsaud M, *et al* (2021) The study of the determinants controlling Arpp19 phosphatase-inhibitory activity reveals an Arpp19/PP2A-B55 feedback loop. *Nat Commun* 12: 3565

Lorca T, Bernis C, Vigneron S, Burgess A, Brioudes E, Labbe JC & Castro A (2010) Constant regulation of both the MPF amplification loop and the Greatwall-PP2A pathway is required for metaphase II arrest and correct entry into the first embryonic cell cycle. *Journal of cell science* 123: 2281–91

Lorca T, Cruzalegui FH, Fesquet D, Cavadore JC, Mery J, Means A & Doree M (1993) Calmodulin-dependent protein kinase II mediates inactivation of MPF and CSF upon fertilization of Xenopus eggs. *Nature* 366: 270–3

Lorca T, Labbe JC, Devault A, Fesquet D, Strausfeld U, Nilsson J, Nygren PA, Uhlen M, Cavadore JC & Doree M (1992) Cyclin A-cdc2 kinase does not trigger but delays cyclin degradation in interphase extracts of amphibian eggs. *Journal of cell science* 102 ( Pt 1): 55–62

Ma S, Vigneron S, Robert P, Strub JM, Cianferani S, Castro A & Lorca T (2016) Greatwall dephosphorylation and inactivation upon mitotic exit is triggered by PP1. *Journal of cell science* 129: 1329–39

Vigneron S, Brioudes E, Burgess A, Labbe JC, Lorca T & Castro A (2009) Greatwall maintains mitosis through regulation of PP2A. *EMBO J* 28: 2786–93

Vigneron S, Gharbi-Ayachi A, Raymond A-A, Burgess A, Labbe J-C, Labesse G, Monsarrat B, Lorca T & Castro A (2011) Characterization of the Mechanisms Controlling Greatwall Activity. *Molecular and Cellular Biology* 31: 2262–2275
