# Supplementary figures and images for: Increases in cyclin A/Cdk activity and in PP2A-B55 inhibition by FAM122A are key mitosis-inducing events (part 1 of 2)
Source: EMBO J. 2024 Feb 20;43(6):993–1014. doi: 10.1038/s44318-024-00054-z (PMC10943098; doi:10.1038/s44318-024-00054-z)

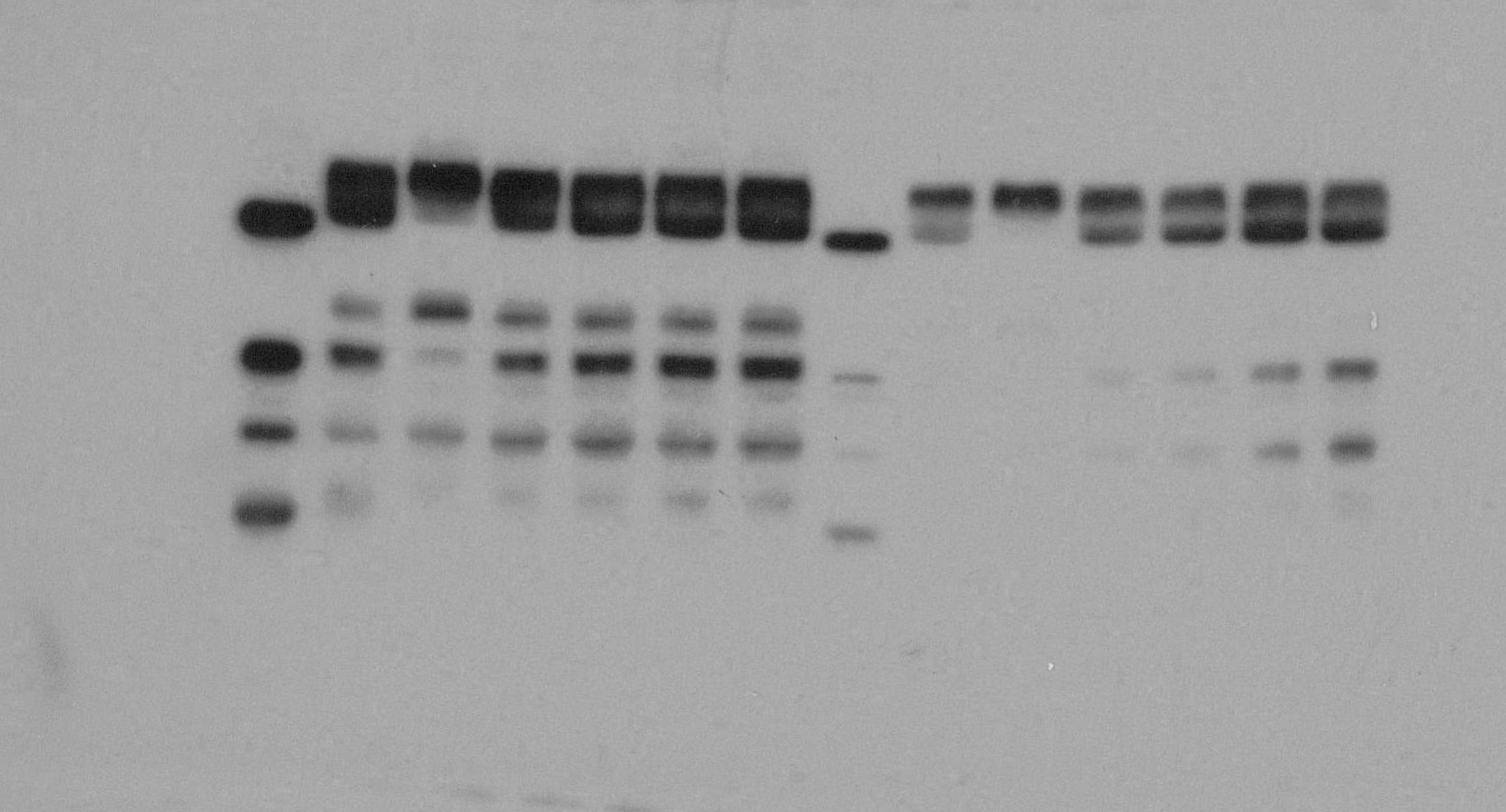

Supplement: Supplementary file 2 — Source Data Fig. 1 [file 44318_2024_54_MOESM2_ESM.zip › Figure 1/Figure 1A/Western 6-His FAM122A.tif]

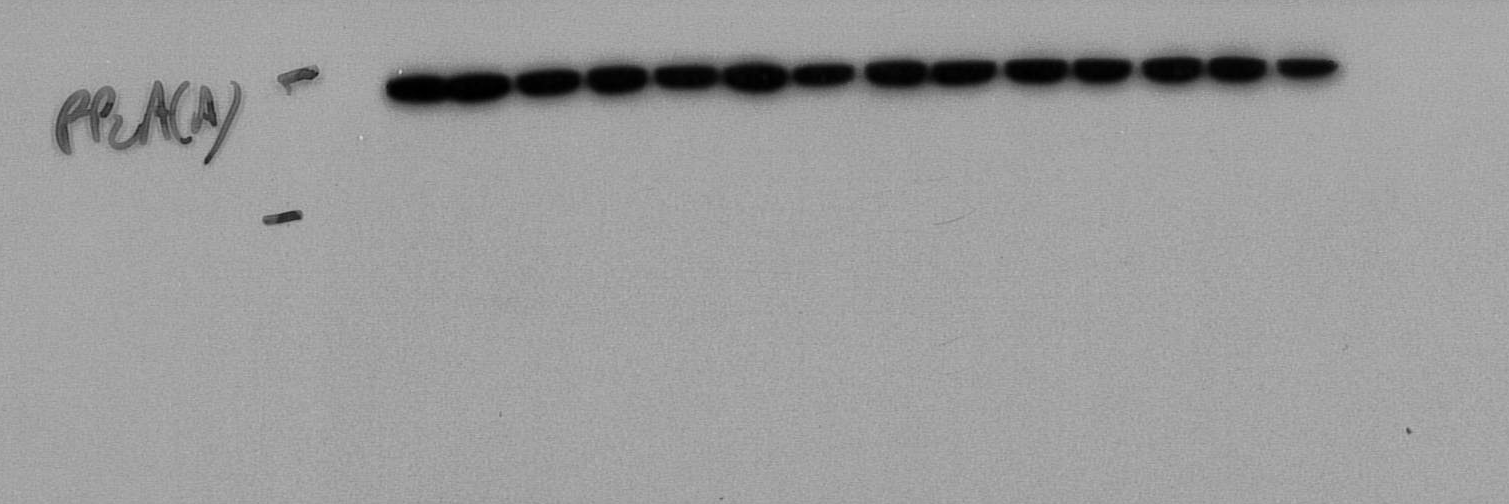

Supplement: Supplementary file 2 — Source Data Fig. 1 [file 44318_2024_54_MOESM2_ESM.zip › Figure 1/Figure 1A/Western PP2A-A-1.tif]

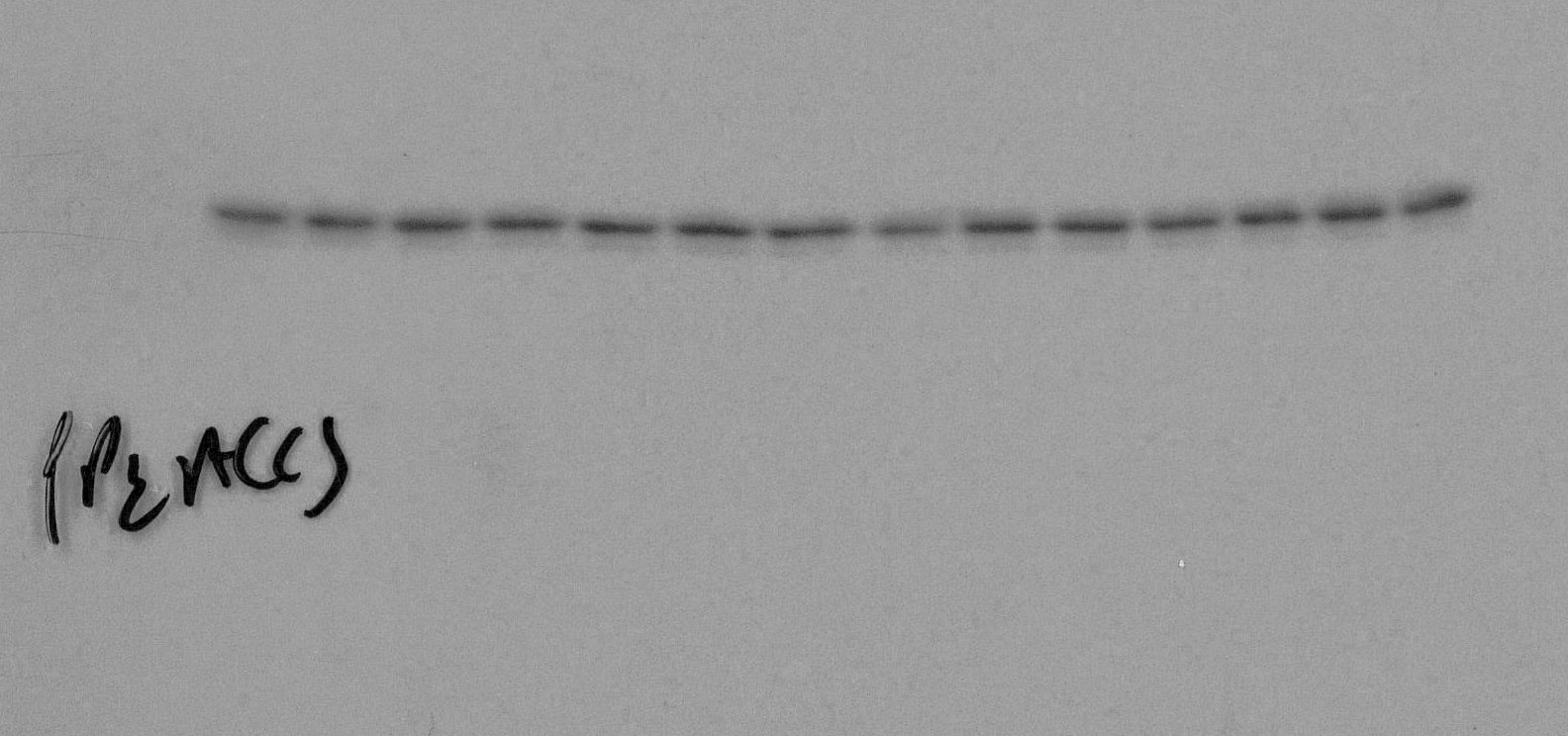

Supplement: Supplementary file 2 — Source Data Fig. 1 [file 44318_2024_54_MOESM2_ESM.zip › Figure 1/Figure 1A/Western PP2A(C).jpg]

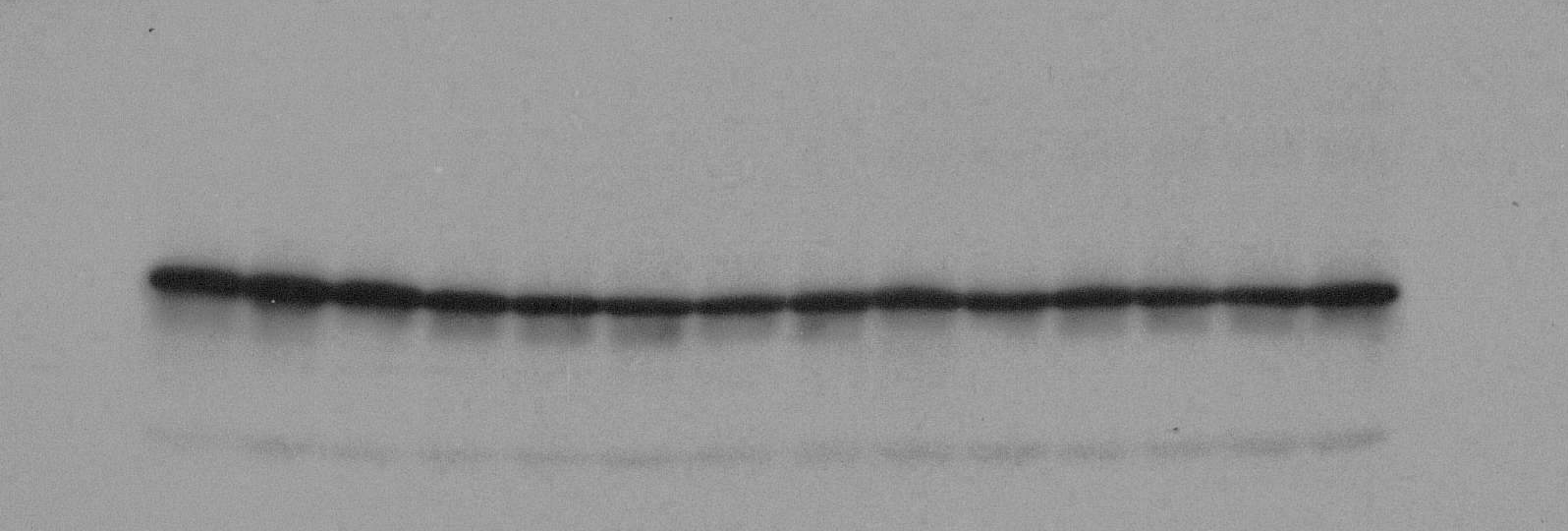

Supplement: Supplementary file 2 — Source Data Fig. 1 [file 44318_2024_54_MOESM2_ESM.zip › Figure 1/Figure 1A/Western Wee1.tif]

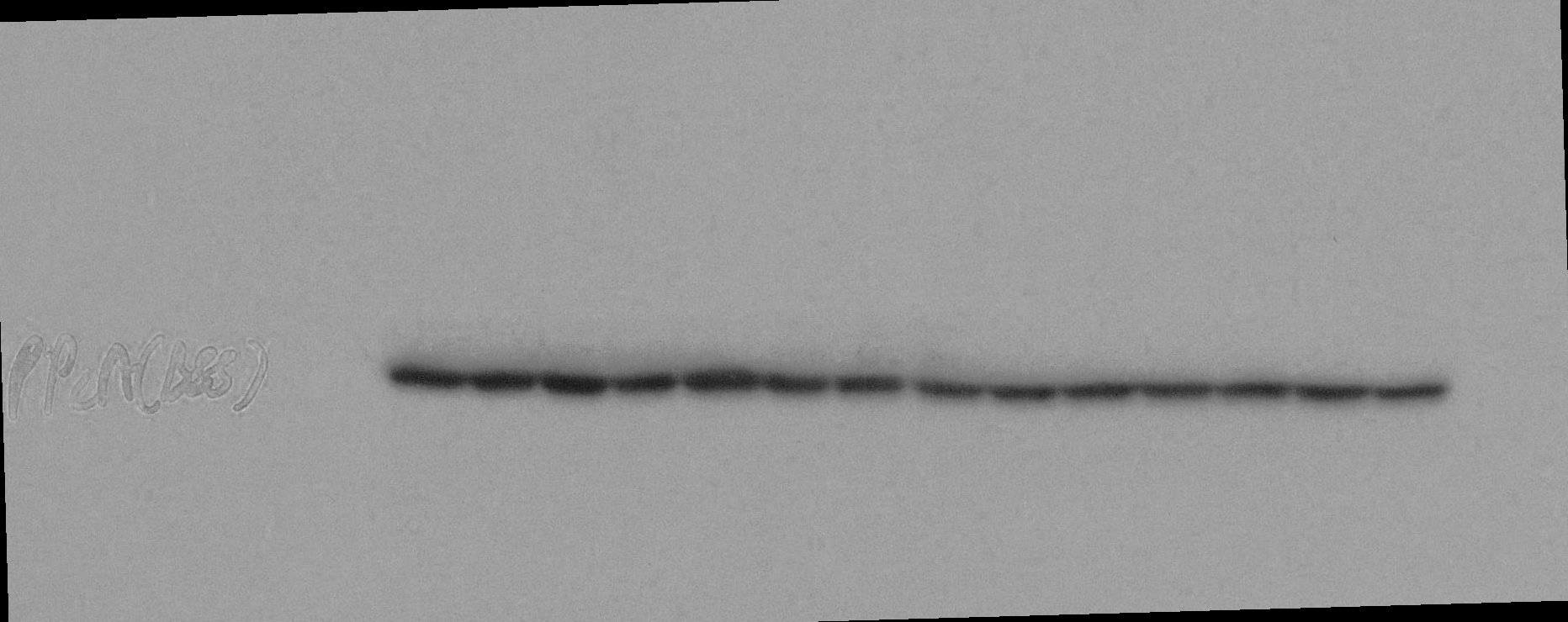

Supplement: Supplementary file 2 — Source Data Fig. 1 [file 44318_2024_54_MOESM2_ESM.zip › Figure 1/Figure 1A/Western PP2A B55.jpg]

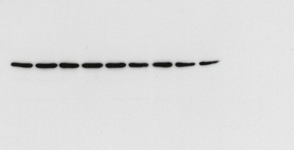

Supplement: Supplementary file 2 — Source Data Fig. 1 [file 44318_2024_54_MOESM2_ESM.zip › Figure 1/Figure 1B Upper panel/Western His-FAM + HuFAM122A.tif]

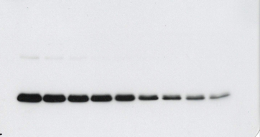

Supplement: Supplementary file 2 — Source Data Fig. 1 [file 44318_2024_54_MOESM2_ESM.zip › Figure 1/Figure 1B Upper panel/western P-S113-Arpp19 + HuFAM122A.tif]

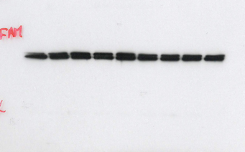

Supplement: Supplementary file 2 — Source Data Fig. 1 [file 44318_2024_54_MOESM2_ESM.zip › Figure 1/Figure 1B Upper panel/Western HIS-FAM + Xe FAM122A.tif]

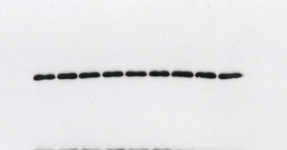

Supplement: Supplementary file 2 — Source Data Fig. 1 [file 44318_2024_54_MOESM2_ESM.zip › Figure 1/Figure 1B Upper panel/Western Arpp19 -FAM122A.tif]

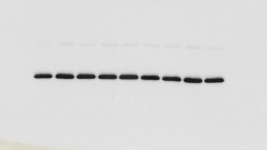

Supplement: Supplementary file 2 — Source Data Fig. 1 [file 44318_2024_54_MOESM2_ESM.zip › Figure 1/Figure 1B Upper panel/Western Arpp19 + Hu FAM122A.tif]

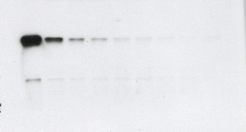

Supplement: Supplementary file 2 — Source Data Fig. 1 [file 44318_2024_54_MOESM2_ESM.zip › Figure 1/Figure 1B Upper panel/Western P-S113 Arpp19 -FAM122A.tif]

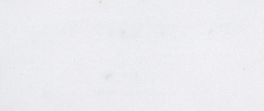

Supplement: Supplementary file 2 — Source Data Fig. 1 [file 44318_2024_54_MOESM2_ESM.zip › Figure 1/Figure 1B Upper panel/Western His-FAM. -FAM122A .tif]

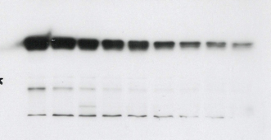

Supplement: Supplementary file 2 — Source Data Fig. 1 [file 44318_2024_54_MOESM2_ESM.zip › Figure 1/Figure 1B Upper panel/Western P-S113 Arpp19 + Xe FAM122A.tif]

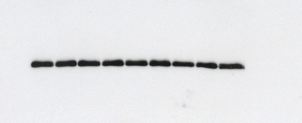

Supplement: Supplementary file 2 — Source Data Fig. 1 [file 44318_2024_54_MOESM2_ESM.zip › Figure 1/Figure 1B Upper panel/Western Arpp19 +Xe FAM122A.tif]

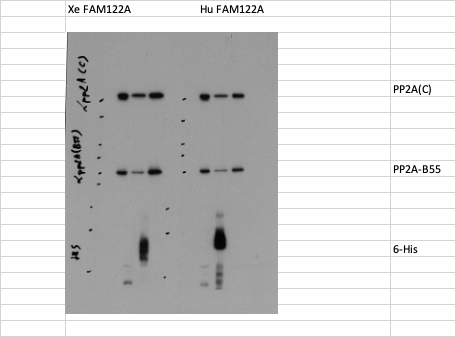

Supplement: Supplementary file 2 — Source Data Fig. 1 [file 44318_2024_54_MOESM2_ESM.zip › Figure 1/Figure 1C/Western PP2A(C), B55 and HIS-FAM.tif]

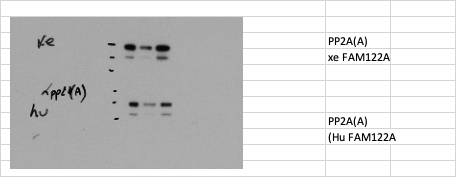

Supplement: Supplementary file 2 — Source Data Fig. 1 [file 44318_2024_54_MOESM2_ESM.zip › Figure 1/Figure 1C/Western PP2A(A).tif]

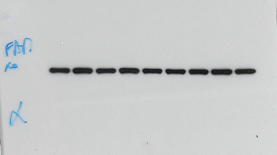

Supplement: Supplementary file 2 — Source Data Fig. 1 [file 44318_2024_54_MOESM2_ESM.zip › Figure 1/Figure 1B Lower Panel/Western PRC1 +Xe FAM122A.tif]

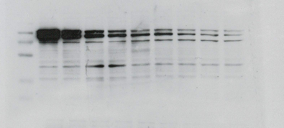

Supplement: Supplementary file 2 — Source Data Fig. 1 [file 44318_2024_54_MOESM2_ESM.zip › Figure 1/Figure 1B Lower Panel/Western PRC1 PT481 +Hu FAM122A.tif]

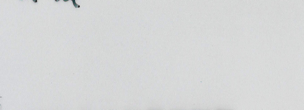

Supplement: Supplementary file 2 — Source Data Fig. 1 [file 44318_2024_54_MOESM2_ESM.zip › Figure 1/Figure 1B Lower Panel/Western HIS-FAM -FAM122A.tif]

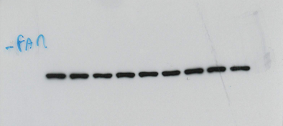

Supplement: Supplementary file 2 — Source Data Fig. 1 [file 44318_2024_54_MOESM2_ESM.zip › Figure 1/Figure 1B Lower Panel/Western PRC1 -FAM122A.tif]

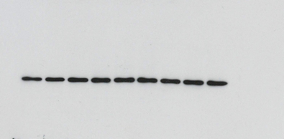

Supplement: Supplementary file 2 — Source Data Fig. 1 [file 44318_2024_54_MOESM2_ESM.zip › Figure 1/Figure 1B Lower Panel/western HIS-FAM + Xe Fam122A.tif]

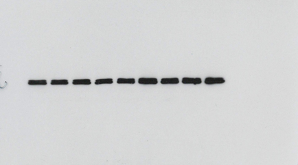

Supplement: Supplementary file 2 — Source Data Fig. 1 [file 44318_2024_54_MOESM2_ESM.zip › Figure 1/Figure 1B Lower Panel/Westren HIS-FAM +Hu FAM122A.tif]

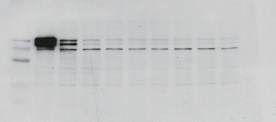

Supplement: Supplementary file 2 — Source Data Fig. 1 [file 44318_2024_54_MOESM2_ESM.zip › Figure 1/Figure 1B Lower Panel/Western PRC1 P-T481 - FAM122A.tif]

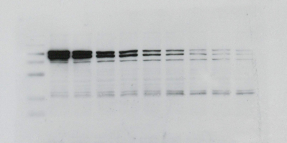

Supplement: Supplementary file 2 — Source Data Fig. 1 [file 44318_2024_54_MOESM2_ESM.zip › Figure 1/Figure 1B Lower Panel/Western PRC1 P-T481 +Xe FAM122A.tif]

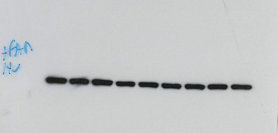

Supplement: Supplementary file 2 — Source Data Fig. 1 [file 44318_2024_54_MOESM2_ESM.zip › Figure 1/Figure 1B Lower Panel/Western PRC1 + Hu FAM122A.tif]

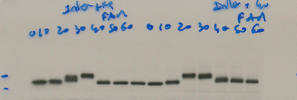

Supplement: Supplementary file 3 — Source Data Fig. 2 [file 44318_2024_54_MOESM3_ESM.zip › Figure 2/Figure 2A/Western Gwl.tif]

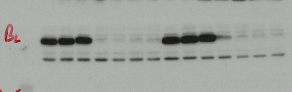

Supplement: Supplementary file 3 — Source Data Fig. 2 [file 44318_2024_54_MOESM3_ESM.zip › Figure 2/Figure 2A/western Cyclin B2.tif]

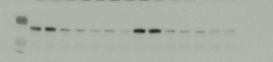

Supplement: Supplementary file 3 — Source Data Fig. 2 [file 44318_2024_54_MOESM3_ESM.zip › Figure 2/Figure 2A/western P-Tyr.tif]

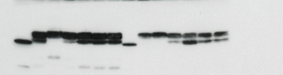

Supplement: Supplementary file 3 — Source Data Fig. 2 [file 44318_2024_54_MOESM3_ESM.zip › Figure 2/Figure 2A/western HIS FAM122A.tif]

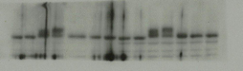

Supplement: Supplementary file 3 — Source Data Fig. 2 [file 44318_2024_54_MOESM3_ESM.zip › Figure 2/Figure 2A/Western Cdc27.tif]

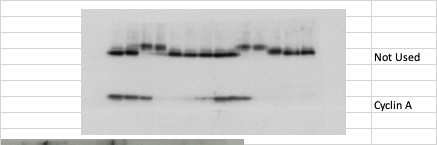

Supplement: Supplementary file 3 — Source Data Fig. 2 [file 44318_2024_54_MOESM3_ESM.zip › Figure 2/Figure 2A/western CycA.tif]

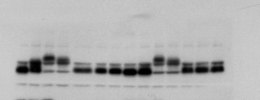

Supplement: Supplementary file 3 — Source Data Fig. 2 [file 44318_2024_54_MOESM3_ESM.zip › Figure 2/Figure 2A/western cdc25.tif]

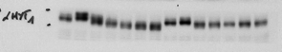

Supplement: Supplementary file 3 — Source Data Fig. 2 [file 44318_2024_54_MOESM3_ESM.zip › Figure 2/Figure 2F left Panel/western Myt1.tif]

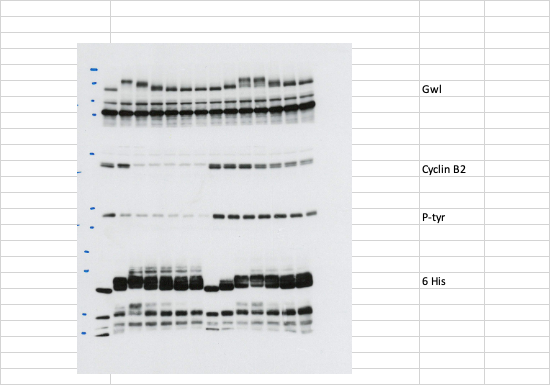

Supplement: Supplementary file 3 — Source Data Fig. 2 [file 44318_2024_54_MOESM3_ESM.zip › Figure 2/Figure 2F left Panel/western Gwl, Cyclin B2, P-Tyr, HIS FAM122A.tif]

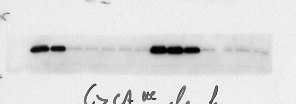

Supplement: Supplementary file 3 — Source Data Fig. 2 [file 44318_2024_54_MOESM3_ESM.zip › Figure 2/Figure 2F left Panel/Western Cyclin A.tif]

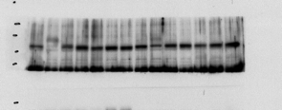

Supplement: Supplementary file 3 — Source Data Fig. 2 [file 44318_2024_54_MOESM3_ESM.zip › Figure 2/Figure 2F left Panel/western Cdc27.tif]

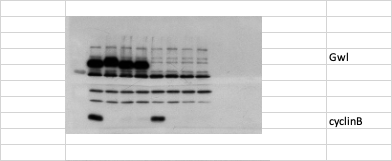

Supplement: Supplementary file 3 — Source Data Fig. 2 [file 44318_2024_54_MOESM3_ESM.zip › Figure 2/Figure 2C/western Gwl and Cyclin B2.tif]

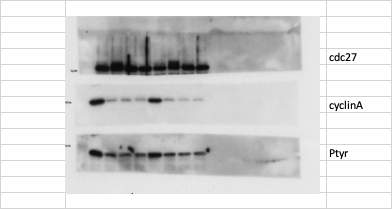

Supplement: Supplementary file 3 — Source Data Fig. 2 [file 44318_2024_54_MOESM3_ESM.zip › Figure 2/Figure 2C/western Cdc27 and Cyclin A and P-tyr.tif]

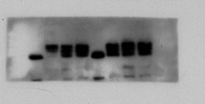

Supplement: Supplementary file 3 — Source Data Fig. 2 [file 44318_2024_54_MOESM3_ESM.zip › Figure 2/Figure 2C/western HIS FAM122A.tif]

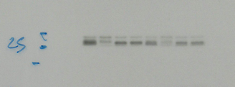

Supplement: Supplementary file 3 — Source Data Fig. 2 [file 44318_2024_54_MOESM3_ESM.zip › Figure 2/Figure 2C/Western Cdc25.tif]

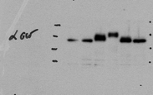

Supplement: Supplementary file 3 — Source Data Fig. 2 [file 44318_2024_54_MOESM3_ESM.zip › Figure 2/Figure 2D/western Gwl.tif]

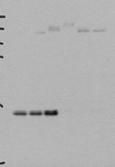

Supplement: Supplementary file 3 — Source Data Fig. 2 [file 44318_2024_54_MOESM3_ESM.zip › Figure 2/Figure 2D/western Cyclin B2.tif]

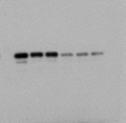

Supplement: Supplementary file 3 — Source Data Fig. 2 [file 44318_2024_54_MOESM3_ESM.zip › Figure 2/Figure 2D/western P-Tyr.tif]

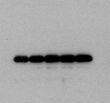

Supplement: Supplementary file 3 — Source Data Fig. 2 [file 44318_2024_54_MOESM3_ESM.zip › Figure 2/Figure 2D/western HIS FAM122A.tif]

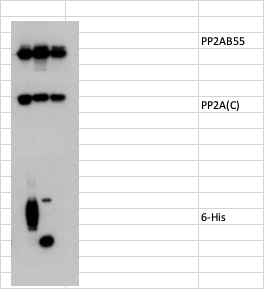

Supplement: Supplementary file 3 — Source Data Fig. 2 [file 44318_2024_54_MOESM3_ESM.zip › Figure 2/Figure 2E/Western PP2A(BB5, (C), HIS FAM122A .tif]

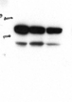

Supplement: Supplementary file 3 — Source Data Fig. 2 [file 44318_2024_54_MOESM3_ESM.zip › Figure 2/Figure 2E/western PP2A(A).tif]

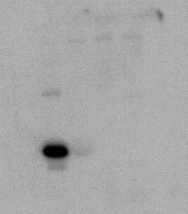

Supplement: Supplementary file 3 — Source Data Fig. 2 [file 44318_2024_54_MOESM3_ESM.zip › Figure 2/Figure 2B/33P-Arpp19.tif]

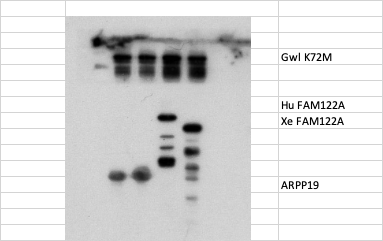

Supplement: Supplementary file 3 — Source Data Fig. 2 [file 44318_2024_54_MOESM3_ESM.zip › Figure 2/Figure 2B/Western Gwk K72M, Hu and Xe FAM 122A Arpp19.tif]

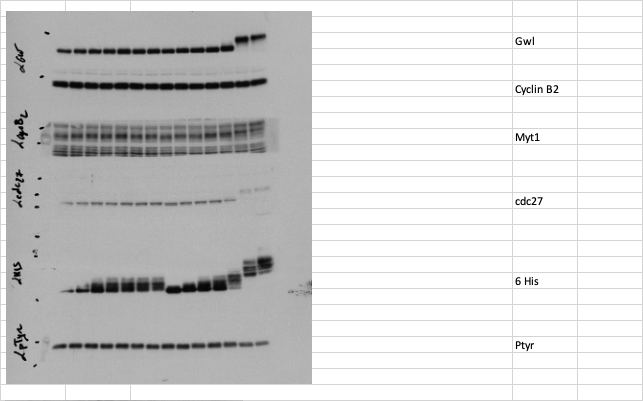

Supplement: Supplementary file 3 — Source Data Fig. 2 [file 44318_2024_54_MOESM3_ESM.zip › Figure 2/Figure 2F Right Panel/Western Gwl, Cyclin B2, Myt1, Cdc27, His FAM, P-Tyr.tif]

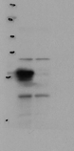

Supplement: Supplementary file 3 — Source Data Fig. 2 [file 44318_2024_54_MOESM3_ESM.zip › Figure 2/Figure 2F Right Panel/western Cyclin A for depletion.tif]

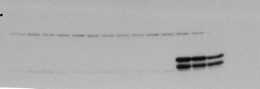

Supplement: Supplementary file 3 — Source Data Fig. 2 [file 44318_2024_54_MOESM3_ESM.zip › Figure 2/Figure 2F Right Panel/western Cyclin A .tif]

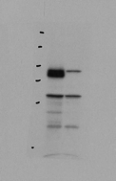

Supplement: Supplementary file 3 — Source Data Fig. 2 [file 44318_2024_54_MOESM3_ESM.zip › Figure 2/Figure 2F Right Panel/western Cdc25.tif]

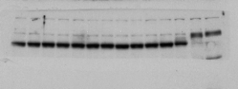

Supplement: Supplementary file 3 — Source Data Fig. 2 [file 44318_2024_54_MOESM3_ESM.zip › Figure 2/Figure 2F Right Panel/western cdc27 high exposure.tif]

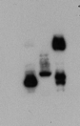

Supplement: Supplementary file 4 — Source Data Fig. 3 [file 44318_2024_54_MOESM4_ESM.zip › Figure 3/Figure 3E/western HIS FAM.tif]

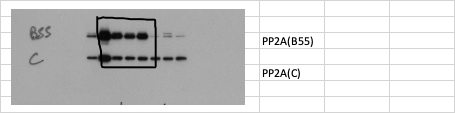

Supplement: Supplementary file 4 — Source Data Fig. 3 [file 44318_2024_54_MOESM4_ESM.zip › Figure 3/Figure 3E/western PP2A(B55) and (C).tif]

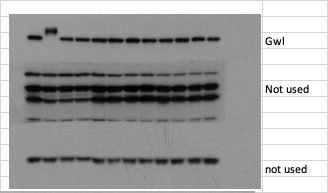

Supplement: Supplementary file 4 — Source Data Fig. 3 [file 44318_2024_54_MOESM4_ESM.zip › Figure 3/Figure 3B/Western Gwl.tif]

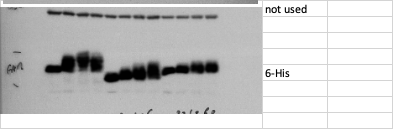

Supplement: Supplementary file 4 — Source Data Fig. 3 [file 44318_2024_54_MOESM4_ESM.zip › Figure 3/Figure 3B/western HIS)FAM122A.tif]

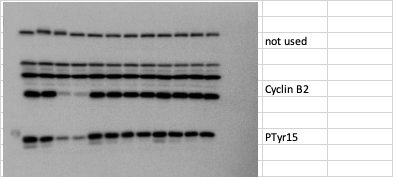

Supplement: Supplementary file 4 — Source Data Fig. 3 [file 44318_2024_54_MOESM4_ESM.zip › Figure 3/Figure 3B/western Cyclin B2 and P-Tyr.tif]

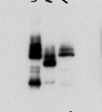

Supplement: Supplementary file 4 — Source Data Fig. 3 [file 44318_2024_54_MOESM4_ESM.zip › Figure 3/Figure 3C/western HIS FAM.tif]

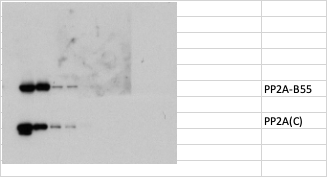

Supplement: Supplementary file 4 — Source Data Fig. 3 [file 44318_2024_54_MOESM4_ESM.zip › Figure 3/Figure 3C/western PP2A(B55) and (C).tif]

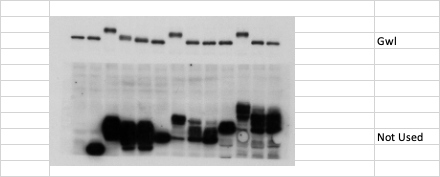

Supplement: Supplementary file 4 — Source Data Fig. 3 [file 44318_2024_54_MOESM4_ESM.zip › Figure 3/Figure 3D/Western Gwl.tif]

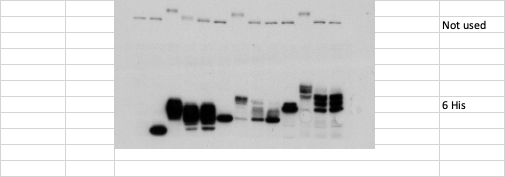

Supplement: Supplementary file 4 — Source Data Fig. 3 [file 44318_2024_54_MOESM4_ESM.zip › Figure 3/Figure 3D/western His FAM122A.tif]

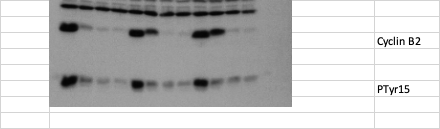

Supplement: Supplementary file 4 — Source Data Fig. 3 [file 44318_2024_54_MOESM4_ESM.zip › Figure 3/Figure 3D/western Cyclin B2 and P-TYR.tif]

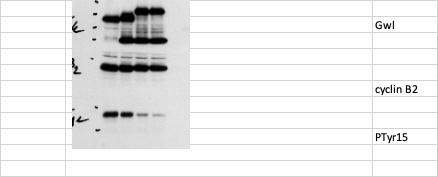

Supplement: Supplementary file 5 — Source Data Fig. 4 [file 44318_2024_54_MOESM5_ESM.zip › Figure 4/Figure 4B Delta CDC27/western gwl, Cyclin B2 and Ptyr.tif]

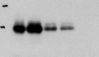

Supplement: Supplementary file 5 — Source Data Fig. 4 [file 44318_2024_54_MOESM5_ESM.zip › Figure 4/Figure 4B Delta CDC27/western PP2AB55.tif]

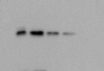

Supplement: Supplementary file 5 — Source Data Fig. 4 [file 44318_2024_54_MOESM5_ESM.zip › Figure 4/Figure 4B Delta CDC27/western PP2A(C).tif]

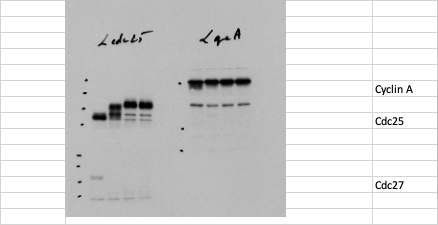

Supplement: Supplementary file 5 — Source Data Fig. 4 [file 44318_2024_54_MOESM5_ESM.zip › Figure 4/Figure 4B Delta CDC27/western cdc25, Cyclin A and cdc27.tif]

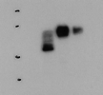

Supplement: Supplementary file 5 — Source Data Fig. 4 [file 44318_2024_54_MOESM5_ESM.zip › Figure 4/Figure 4B Delta CDC27/western HIS-FAM.tif]

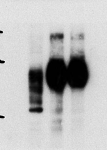

Supplement: Supplementary file 5 — Source Data Fig. 4 [file 44318_2024_54_MOESM5_ESM.zip › Figure 4/Figure 4B Delta CDC27/western His FAM122A.tif]

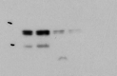

Supplement: Supplementary file 5 — Source Data Fig. 4 [file 44318_2024_54_MOESM5_ESM.zip › Figure 4/Figure 4B Delta CDC27/western PP2A(A).tif]

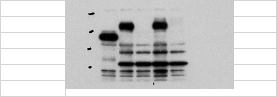

Supplement: Supplementary file 5 — Source Data Fig. 4 [file 44318_2024_54_MOESM5_ESM.zip › Figure 4/Figure 4C Left panel/western Gwl.tif]

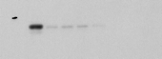

Supplement: Supplementary file 5 — Source Data Fig. 4 [file 44318_2024_54_MOESM5_ESM.zip › Figure 4/Figure 4C Left panel/western PTyr.tif]

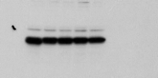

Supplement: Supplementary file 5 — Source Data Fig. 4 [file 44318_2024_54_MOESM5_ESM.zip › Figure 4/Figure 4C Left panel/Western Cyclin B.tif]

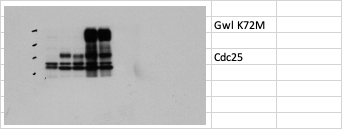

Supplement: Supplementary file 5 — Source Data Fig. 4 [file 44318_2024_54_MOESM5_ESM.zip › Figure 4/Figure 4C Left panel/western GwlK72M .tif]

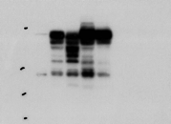

Supplement: Supplementary file 5 — Source Data Fig. 4 [file 44318_2024_54_MOESM5_ESM.zip › Figure 4/Figure 4C Left panel/western HIS FAM122A.tif]

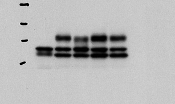

Supplement: Supplementary file 5 — Source Data Fig. 4 [file 44318_2024_54_MOESM5_ESM.zip › Figure 4/Figure 4C Left panel/western Cdc25.tif]

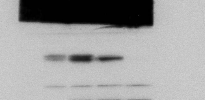

Supplement: Supplementary file 5 — Source Data Fig. 4 [file 44318_2024_54_MOESM5_ESM.zip › Figure 4/Figure 4B delta control /western Cyclin B2.tif]

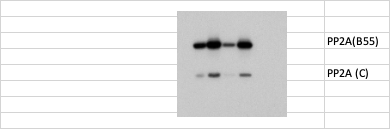

Supplement: Supplementary file 5 — Source Data Fig. 4 [file 44318_2024_54_MOESM5_ESM.zip › Figure 4/Figure 4B delta control /western PP2A(B55) and (C).tif]

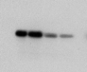

Supplement: Supplementary file 5 — Source Data Fig. 4 [file 44318_2024_54_MOESM5_ESM.zip › Figure 4/Figure 4B delta control /Western PTyr .tif]

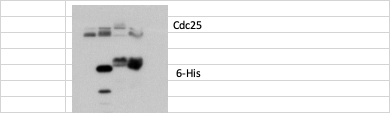

Supplement: Supplementary file 5 — Source Data Fig. 4 [file 44318_2024_54_MOESM5_ESM.zip › Figure 4/Figure 4B delta control /western Cdc25 and HIS FAM l.tif]

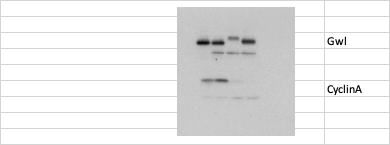

Supplement: Supplementary file 5 — Source Data Fig. 4 [file 44318_2024_54_MOESM5_ESM.zip › Figure 4/Figure 4B delta control /western Gwl and Cyclin A .tif]

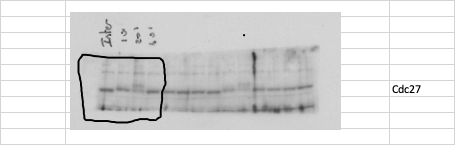

Supplement: Supplementary file 5 — Source Data Fig. 4 [file 44318_2024_54_MOESM5_ESM.zip › Figure 4/Figure 4B delta control /western cdc27.tif]

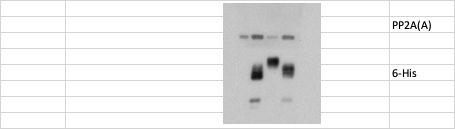

Supplement: Supplementary file 5 — Source Data Fig. 4 [file 44318_2024_54_MOESM5_ESM.zip › Figure 4/Figure 4B delta control /Western PP2A(A) and His FAM122A.tif]

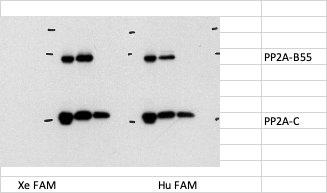

Supplement: Supplementary file 5 — Source Data Fig. 4 [file 44318_2024_54_MOESM5_ESM.zip › Figure 4/Figure 4A/Western PP2A(B55) and (C).tif]

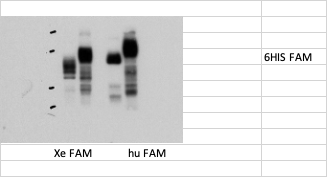

Supplement: Supplementary file 5 — Source Data Fig. 4 [file 44318_2024_54_MOESM5_ESM.zip › Figure 4/Figure 4A/western HIS-FAM122A.tif]

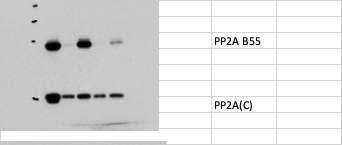

Supplement: Supplementary file 5 — Source Data Fig. 4 [file 44318_2024_54_MOESM5_ESM.zip › Figure 4/Figure 4C Right panel/western PP2AB55 and (C).tif]

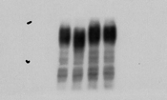

Supplement: Supplementary file 5 — Source Data Fig. 4 [file 44318_2024_54_MOESM5_ESM.zip › Figure 4/Figure 4C Right panel/western HIS FAM 122A.tif]

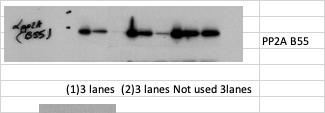

Supplement: Supplementary file 6 — Source Data Fig. 5 [file 44318_2024_54_MOESM6_ESM.zip › Figure 5/Figure 5A/western PP2AB55.tif]

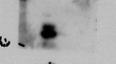

Supplement: Supplementary file 6 — Source Data Fig. 5 [file 44318_2024_54_MOESM6_ESM.zip › Figure 5/Figure 5A/western Arpp19.tif]

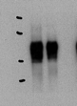

Supplement: Supplementary file 6 — Source Data Fig. 5 [file 44318_2024_54_MOESM6_ESM.zip › Figure 5/Figure 5A/western HIS FAM122A.tif]

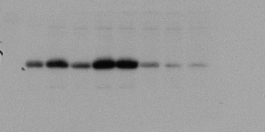

Supplement: Supplementary file 6 — Source Data Fig. 5 [file 44318_2024_54_MOESM6_ESM.zip › Figure 5/Figure 5D/Western Phospho S71 Arpp19.tif]

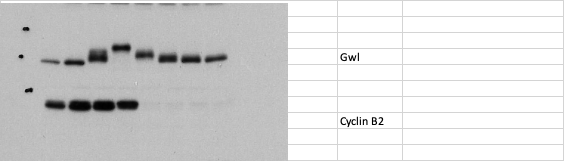

Supplement: Supplementary file 6 — Source Data Fig. 5 [file 44318_2024_54_MOESM6_ESM.zip › Figure 5/Figure 5D/Western Gwl and Cyclin B2.tif]

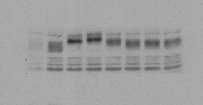

Supplement: Supplementary file 6 — Source Data Fig. 5 [file 44318_2024_54_MOESM6_ESM.zip › Figure 5/Figure 5D/western Myt1.tif]

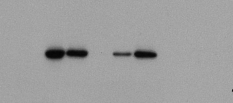

Supplement: Supplementary file 6 — Source Data Fig. 5 [file 44318_2024_54_MOESM6_ESM.zip › Figure 5/Figure 5D/Western PP2A-B55 Left panel.tif]

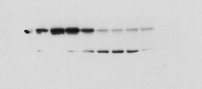

Supplement: Supplementary file 6 — Source Data Fig. 5 [file 44318_2024_54_MOESM6_ESM.zip › Figure 5/Figure 5D/western Cyclin A.tif]

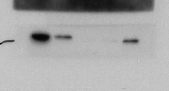

Supplement: Supplementary file 6 — Source Data Fig. 5 [file 44318_2024_54_MOESM6_ESM.zip › Figure 5/Figure 5D/Westren PP2A(A) left panel.tif]

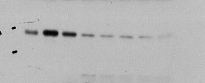

Supplement: Supplementary file 6 — Source Data Fig. 5 [file 44318_2024_54_MOESM6_ESM.zip › Figure 5/Figure 5D/western P-TYR.tif]

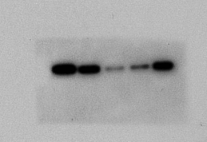

Supplement: Supplementary file 6 — Source Data Fig. 5 [file 44318_2024_54_MOESM6_ESM.zip › Figure 5/Figure 5D/westren PP2A(C) left panel.tif]

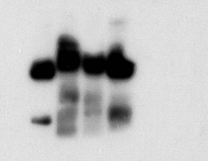

Supplement: Supplementary file 6 — Source Data Fig. 5 [file 44318_2024_54_MOESM6_ESM.zip › Figure 5/Figure 5D/Western HIS FAM 122A.tif]

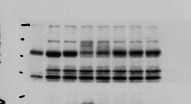

Supplement: Supplementary file 6 — Source Data Fig. 5 [file 44318_2024_54_MOESM6_ESM.zip › Figure 5/Figure 5D/western Cdc27.tif]
