# Supplementary material for: Increases in cyclin A/Cdk activity and in PP2A-B55 inhibition by FAM122A are key mitosis-inducing events
Source: EMBO J. 2024 Feb 20;43(6):993–1014. doi: 10.1038/s44318-024-00054-z (PMC10943098; doi:10.1038/s44318-024-00054-z)
Supplement: Supplementary file 4 — Source Data Fig. 3 [file 44318_2024_54_MOESM4_ESM.zip › Figure 3/Comments for figure 3.docx]

The samples taken as a function of time are those shown and depicted in figure 3.
