# Supplementary figures and images for: Increases in cyclin A/Cdk activity and in PP2A-B55 inhibition by FAM122A are key mitosis-inducing events (part 2 of 2)
Source: EMBO J. 2024 Feb 20;43(6):993–1014. doi: 10.1038/s44318-024-00054-z (PMC10943098; doi:10.1038/s44318-024-00054-z)

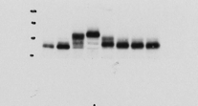

Supplement: Supplementary file 6 — Source Data Fig. 5 [file 44318_2024_54_MOESM6_ESM.zip › Figure 5/Figure 5D/western Cdc25.tif]

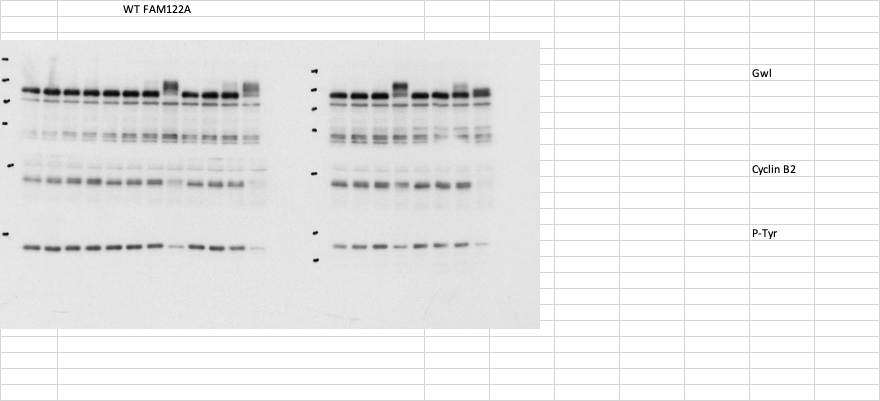

Supplement: Supplementary file 6 — Source Data Fig. 5 [file 44318_2024_54_MOESM6_ESM.zip › Figure 5/Figure 5E/western Gwl, Cyclin B2 and PTyr, WT.tif]

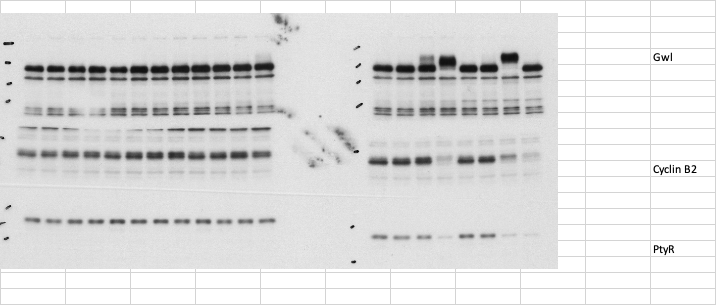

Supplement: Supplementary file 6 — Source Data Fig. 5 [file 44318_2024_54_MOESM6_ESM.zip › Figure 5/Figure 5E/Western Gwl, Cyclin B2 and Ptyr Mutant S:T-A.tif]

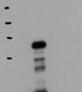

Supplement: Supplementary file 6 — Source Data Fig. 5 [file 44318_2024_54_MOESM6_ESM.zip › Figure 5/Figure 5B/western HIS FAM122A.tif]

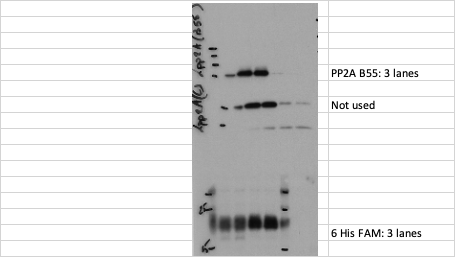

Supplement: Supplementary file 6 — Source Data Fig. 5 [file 44318_2024_54_MOESM6_ESM.zip › Figure 5/Figure 5B/Western PP2AB55 and HIS FAM.tif]

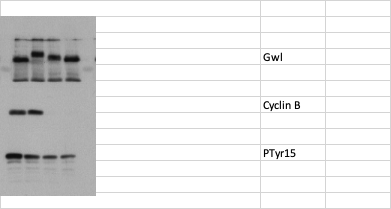

Supplement: Supplementary file 7 — Source Data Fig. 7 [file 44318_2024_54_MOESM7_ESM.zip › Figure 7/Figure 7B/western Gwl, Cyclin B2 and PTyr.tif]

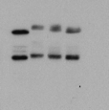

Supplement: Supplementary file 7 — Source Data Fig. 7 [file 44318_2024_54_MOESM7_ESM.zip › Figure 7/Figure 7B/His FAM122A C.E.tif]

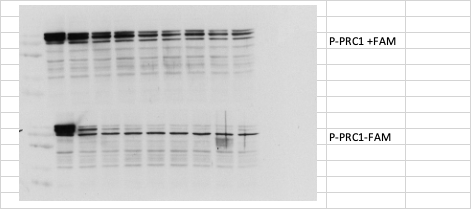

Supplement: Supplementary file 7 — Source Data Fig. 7 [file 44318_2024_54_MOESM7_ESM.zip › Figure 7/Figure 7A/Western p-T481 PRC1 plus and minus FAM 122A.tif]

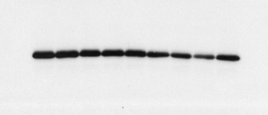

Supplement: Supplementary file 7 — Source Data Fig. 7 [file 44318_2024_54_MOESM7_ESM.zip › Figure 7/Figure 7A/Western Arpp19 Minus FAM 122A.tif]

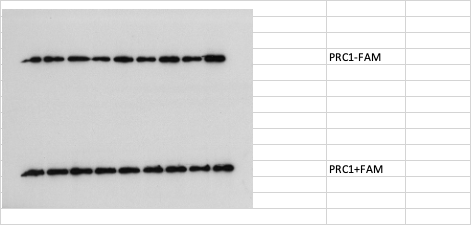

Supplement: Supplementary file 7 — Source Data Fig. 7 [file 44318_2024_54_MOESM7_ESM.zip › Figure 7/Figure 7A/western PRC1 minus or plus FAM122A.tif]

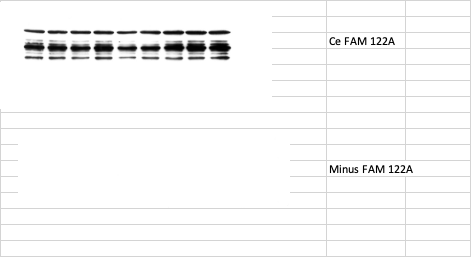

Supplement: Supplementary file 7 — Source Data Fig. 7 [file 44318_2024_54_MOESM7_ESM.zip › Figure 7/Figure 7A/Western HIs-FAM .tif]

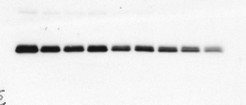

Supplement: Supplementary file 7 — Source Data Fig. 7 [file 44318_2024_54_MOESM7_ESM.zip › Figure 7/Figure 7A/Western P-S113 Arpp19 plus FAM122A.tif]

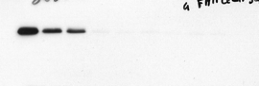

Supplement: Supplementary file 7 — Source Data Fig. 7 [file 44318_2024_54_MOESM7_ESM.zip › Figure 7/Figure 7A/western P-113 ARPP19 Minus FAM122A.tif]

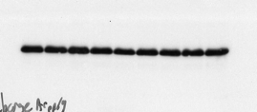

Supplement: Supplementary file 7 — Source Data Fig. 7 [file 44318_2024_54_MOESM7_ESM.zip › Figure 7/Figure 7A/Western Arpp19 Plus FAM 122A.tif]

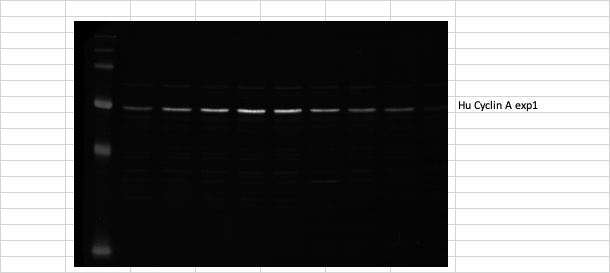

Supplement: Supplementary file 8 — Source Data Fig. 8 [file 44318_2024_54_MOESM8_ESM.zip › Figure 8/Figure 8D/Westren cyclin A experiment 1.tif]

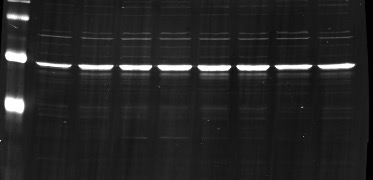

Supplement: Supplementary file 8 — Source Data Fig. 8 [file 44318_2024_54_MOESM8_ESM.zip › Figure 8/Figure 8D/western Cyclin A experiment 2.tif]

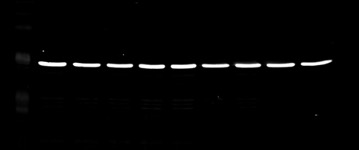

Supplement: Supplementary file 8 — Source Data Fig. 8 [file 44318_2024_54_MOESM8_ESM.zip › Figure 8/Figure 8D/Western Tubulin Experiment 1.tif]

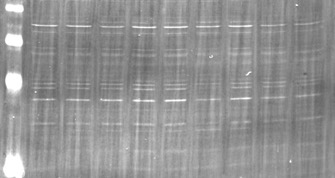

Supplement: Supplementary file 8 — Source Data Fig. 8 [file 44318_2024_54_MOESM8_ESM.zip › Figure 8/Figure 8D/western FAM122A experiment 1.tif]

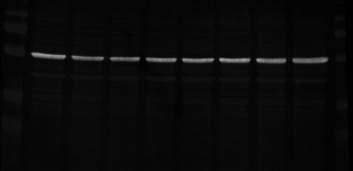

Supplement: Supplementary file 8 — Source Data Fig. 8 [file 44318_2024_54_MOESM8_ESM.zip › Figure 8/Figure 8D/western Tubulin experiment 2.tif]

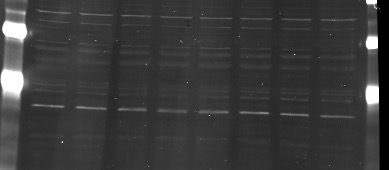

Supplement: Supplementary file 8 — Source Data Fig. 8 [file 44318_2024_54_MOESM8_ESM.zip › Figure 8/Figure 8D/western FAM122A experiment 2.tif]

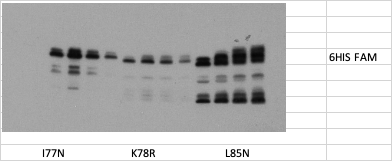

Supplement: Supplementary file 9 — Source Data Appendix Fig. S1_S6 [file 44318_2024_54_MOESM9_ESM.zip › Supp. Figure 1/Supp. Figure 1a upper panel/western HIS FAM upper middle panel.tif]

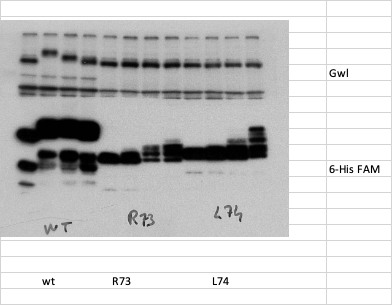

Supplement: Supplementary file 9 — Source Data Appendix Fig. S1_S6 [file 44318_2024_54_MOESM9_ESM.zip › Supp. Figure 1/Supp. Figure 1a upper panel/western Gwl and HIS FAM Upper and left panel .tif]

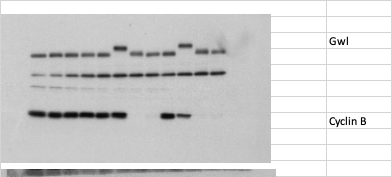

Supplement: Supplementary file 9 — Source Data Appendix Fig. S1_S6 [file 44318_2024_54_MOESM9_ESM.zip › Supp. Figure 1/Supp. Figure 1a upper panel/western Gwl and Cyclin B2 middle upper panel.tif]

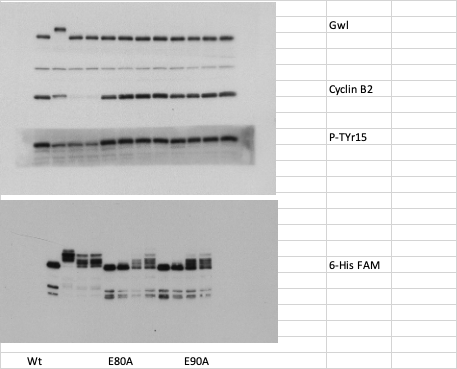

Supplement: Supplementary file 9 — Source Data Appendix Fig. S1_S6 [file 44318_2024_54_MOESM9_ESM.zip › Supp. Figure 1/Supp. Figure 1a upper panel/western Gwl, Cyclin B2, Ptyr, and HisFAM Upper right panel.tif]

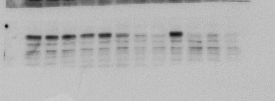

Supplement: Supplementary file 9 — Source Data Appendix Fig. S1_S6 [file 44318_2024_54_MOESM9_ESM.zip › Supp. Figure 1/Supp. Figure 1a upper panel/westren Ptyr Upper middle panel.tif]

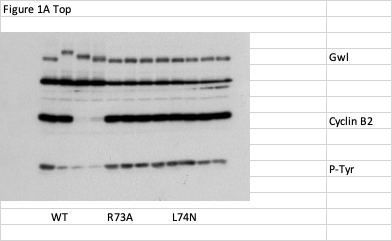

Supplement: Supplementary file 9 — Source Data Appendix Fig. S1_S6 [file 44318_2024_54_MOESM9_ESM.zip › Supp. Figure 1/Supp. Figure 1a upper panel/western Gwl, Cyclin B2 and PTyr, Upper and Left panel.tif]

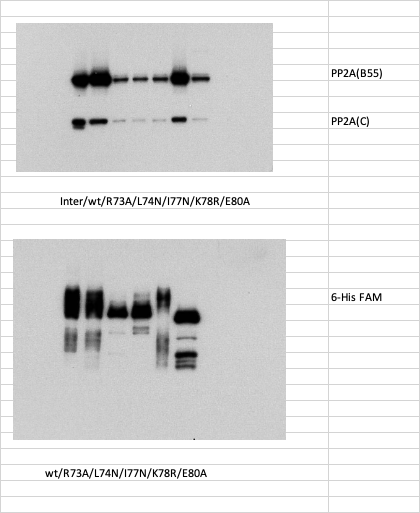

Supplement: Supplementary file 9 — Source Data Appendix Fig. S1_S6 [file 44318_2024_54_MOESM9_ESM.zip › Supp. Figure 1/Supp. Figure 1b/western PP2A(B55), (C) and His FAM left panel.tif]

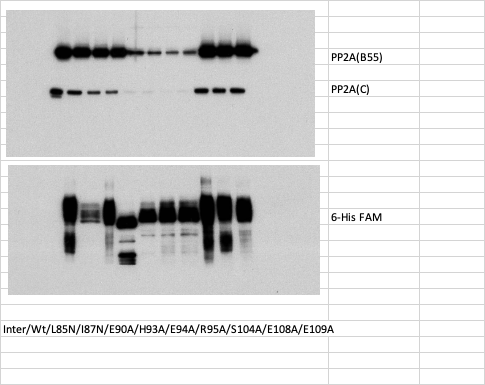

Supplement: Supplementary file 9 — Source Data Appendix Fig. S1_S6 [file 44318_2024_54_MOESM9_ESM.zip › Supp. Figure 1/Supp. Figure 1b/western PP2A(B55), (C┬░ and His FAM Right panel.tif]

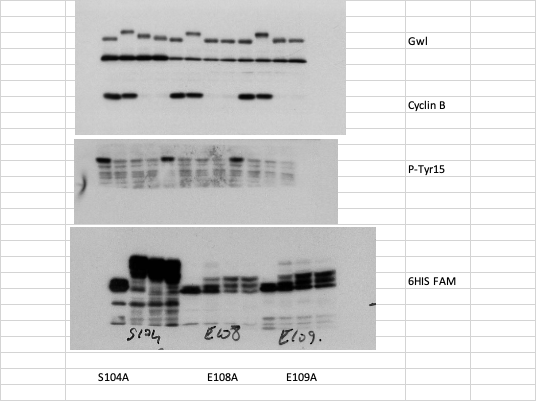

Supplement: Supplementary file 9 — Source Data Appendix Fig. S1_S6 [file 44318_2024_54_MOESM9_ESM.zip › Supp. Figure 1/Supp. Figure 1a Lower panel/western Gwl, Cyclin B2, PTyr and His FAM Right panel.tif]

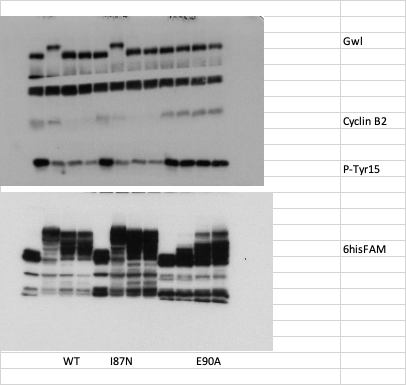

Supplement: Supplementary file 9 — Source Data Appendix Fig. S1_S6 [file 44318_2024_54_MOESM9_ESM.zip › Supp. Figure 1/Supp. Figure 1a Lower panel/Western Gwl, Cyclin B2, Ptyr, His FAM left panel.tif]

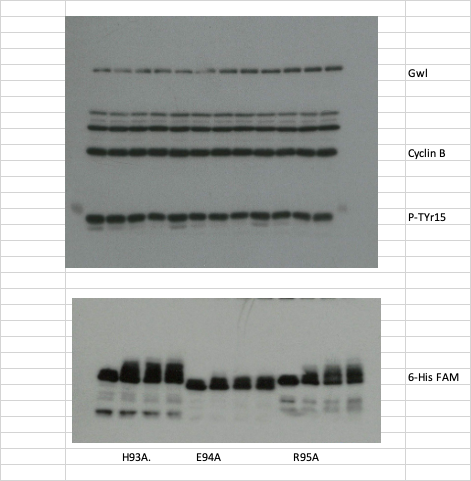

Supplement: Supplementary file 9 — Source Data Appendix Fig. S1_S6 [file 44318_2024_54_MOESM9_ESM.zip › Supp. Figure 1/Supp. Figure 1a Lower panel/western Gwl, Cyclin B2, Ptyr and His FAM Middle panel.tif]

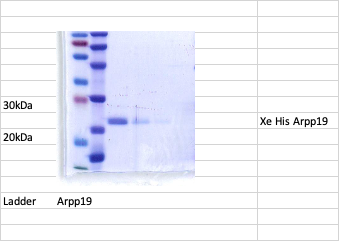

Supplement: Supplementary file 9 — Source Data Appendix Fig. S1_S6 [file 44318_2024_54_MOESM9_ESM.zip › Supp. Figure 2/Supp. Figure 2a/coomacie Xe His Arpp19.tif]

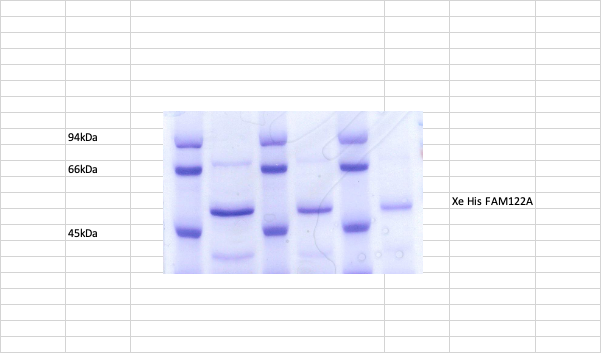

Supplement: Supplementary file 9 — Source Data Appendix Fig. S1_S6 [file 44318_2024_54_MOESM9_ESM.zip › Supp. Figure 2/suppFigure 2c/coomacie supp Figure 2c.tif]

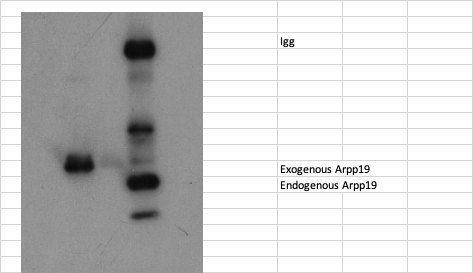

Supplement: Supplementary file 9 — Source Data Appendix Fig. S1_S6 [file 44318_2024_54_MOESM9_ESM.zip › Supp. Figure 2/Supp. Figure 2b/Western Arpp19.tif]

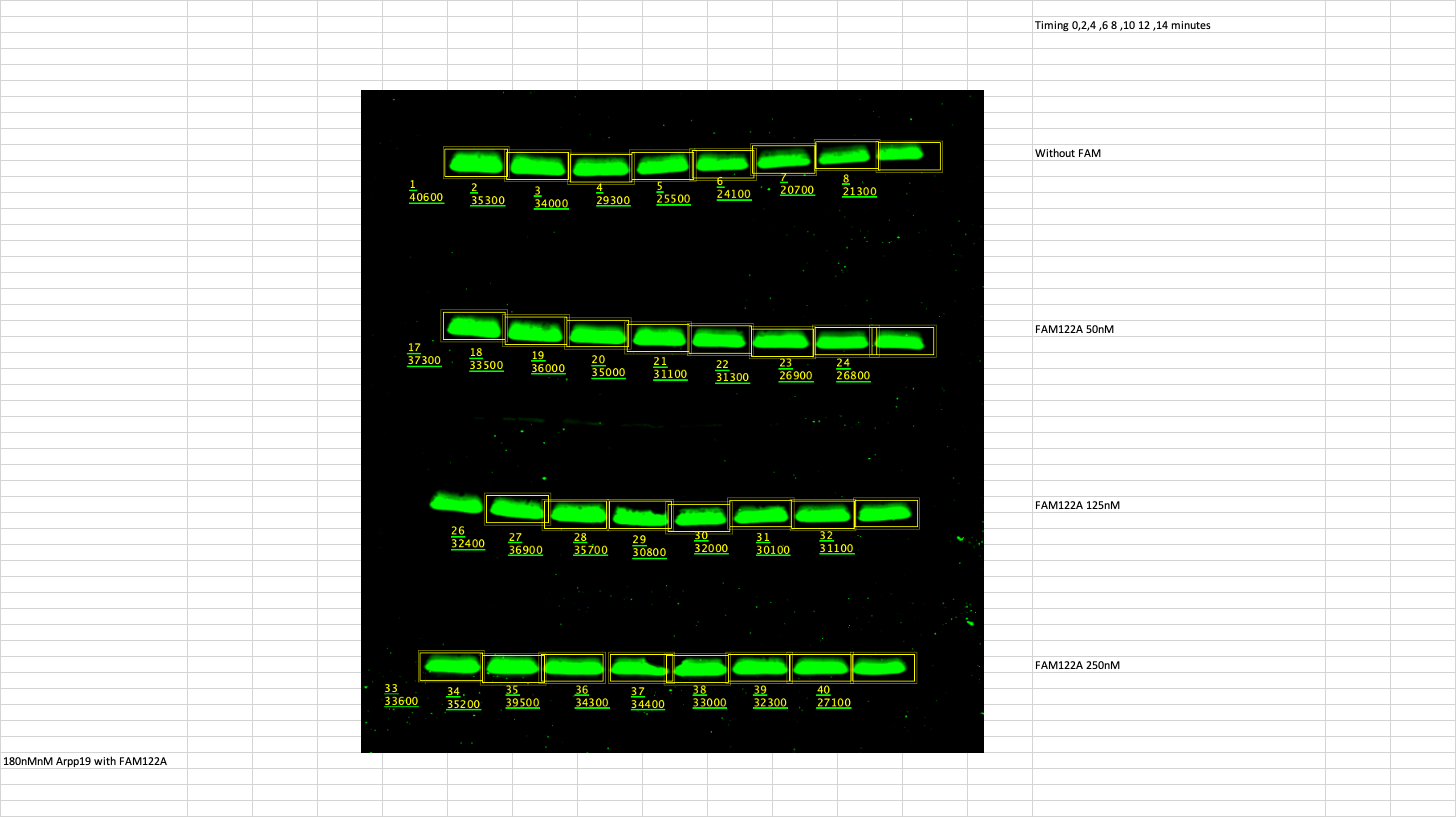

Supplement: Supplementary file 9 — Source Data Appendix Fig. S1_S6 [file 44318_2024_54_MOESM9_ESM.zip › Supp. Figure 3/supp. Figure 3a/western S113-P Arpp19 1200nM +FAM.tif]

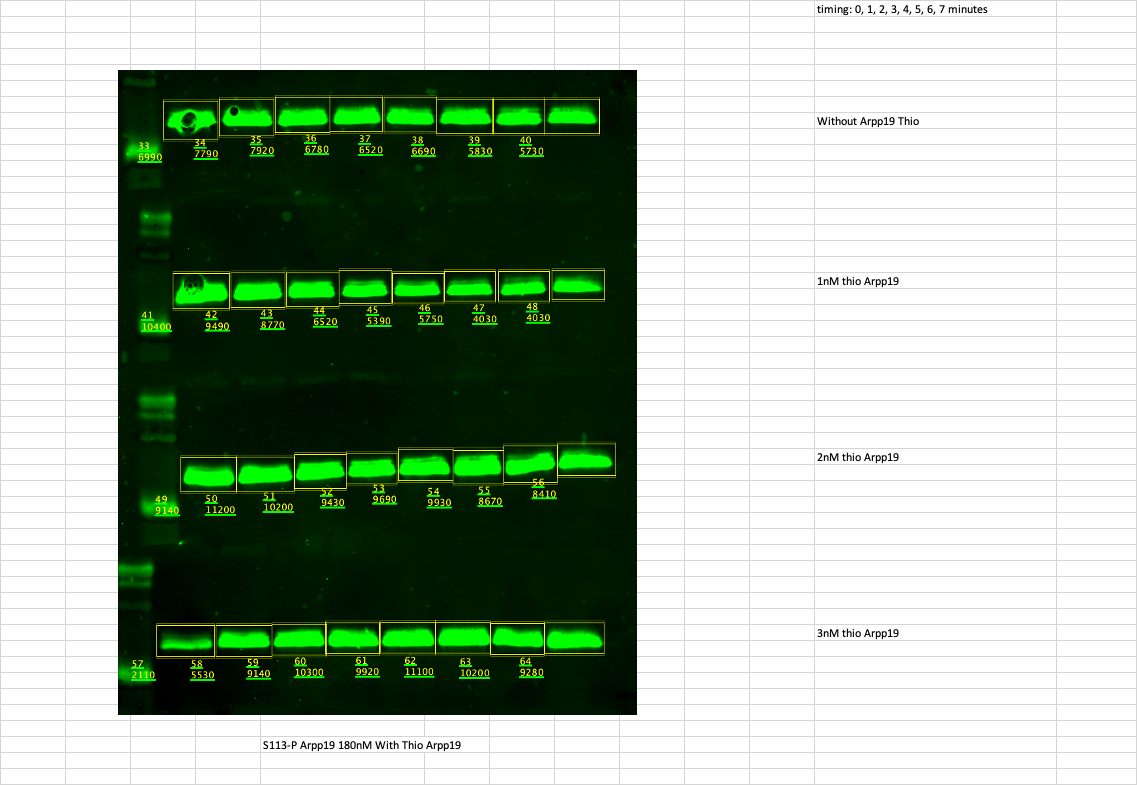

Supplement: Supplementary file 9 — Source Data Appendix Fig. S1_S6 [file 44318_2024_54_MOESM9_ESM.zip › Supp. Figure 3/supp. Figure 3a/WesternS113-P Arpp19 180nM + Thio Arpp19.tif]

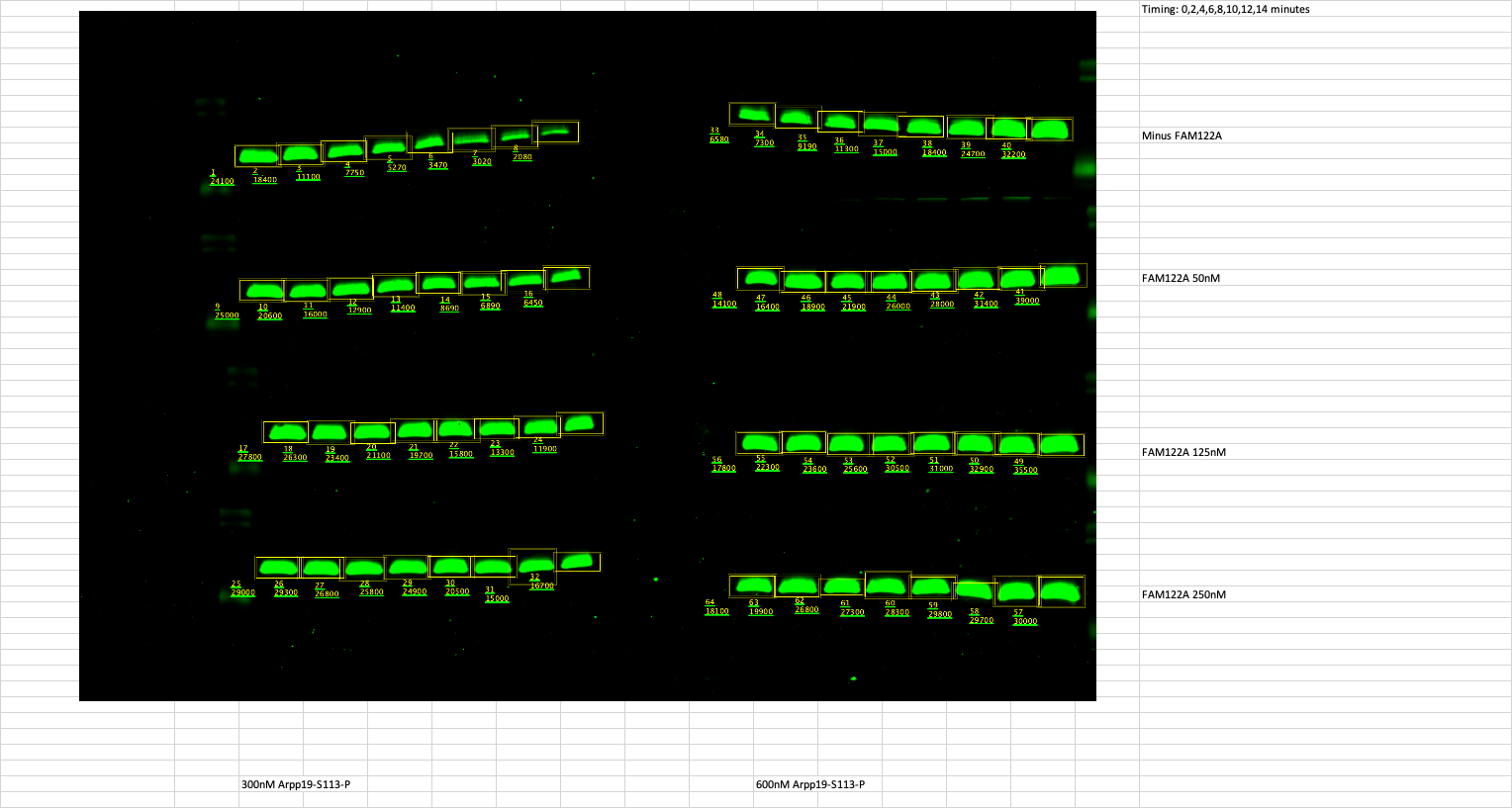

Supplement: Supplementary file 9 — Source Data Appendix Fig. S1_S6 [file 44318_2024_54_MOESM9_ESM.zip › Supp. Figure 3/supp. Figure 3a/western S-113-P Arpp19 300nM and 600nM +FAM.tif]

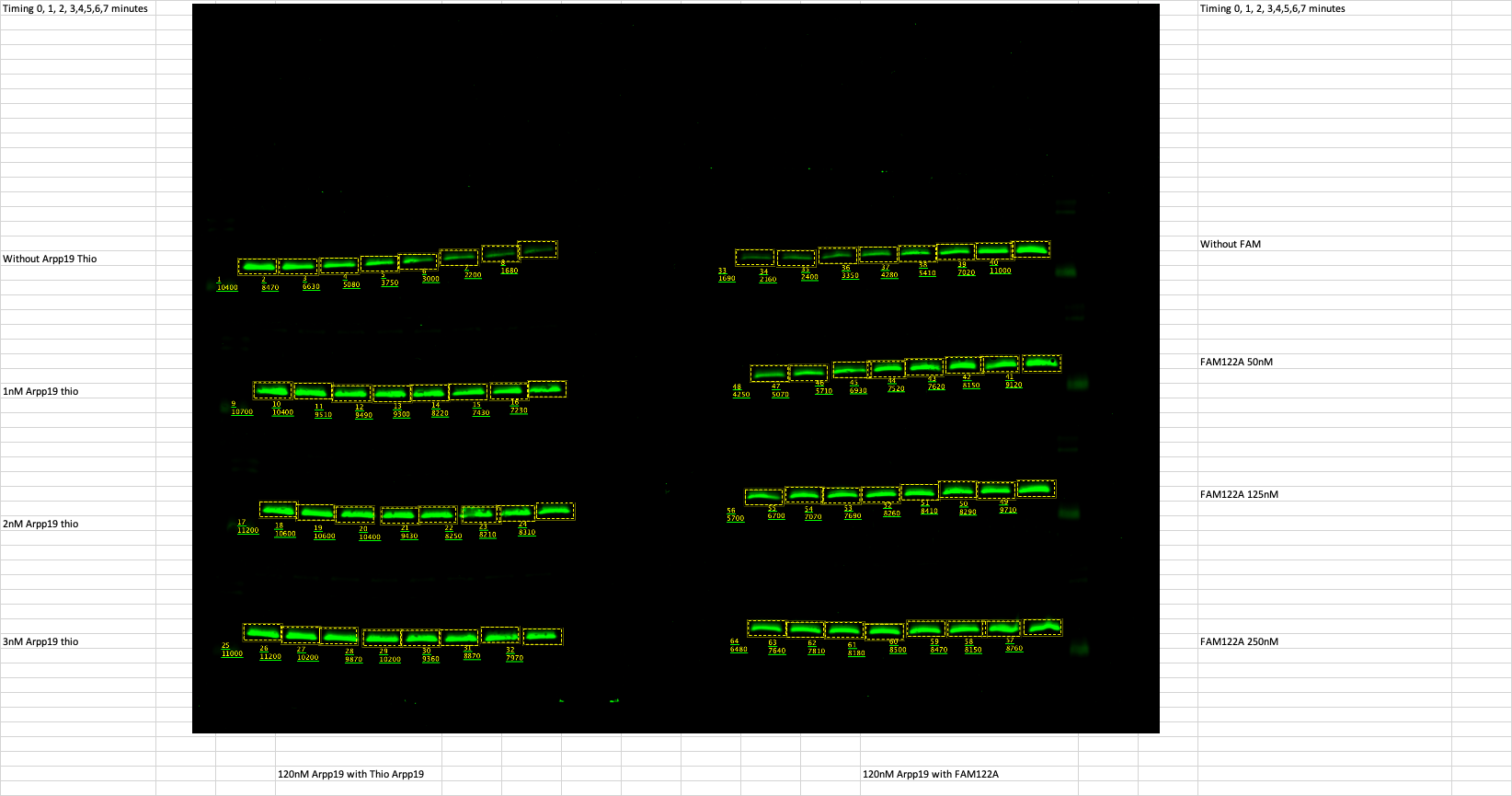

Supplement: Supplementary file 9 — Source Data Appendix Fig. S1_S6 [file 44318_2024_54_MOESM9_ESM.zip › Supp. Figure 3/supp. Figure 3a/Western S113-P Arpp19 120nM +FAM or Arpp19 Thio.tif]

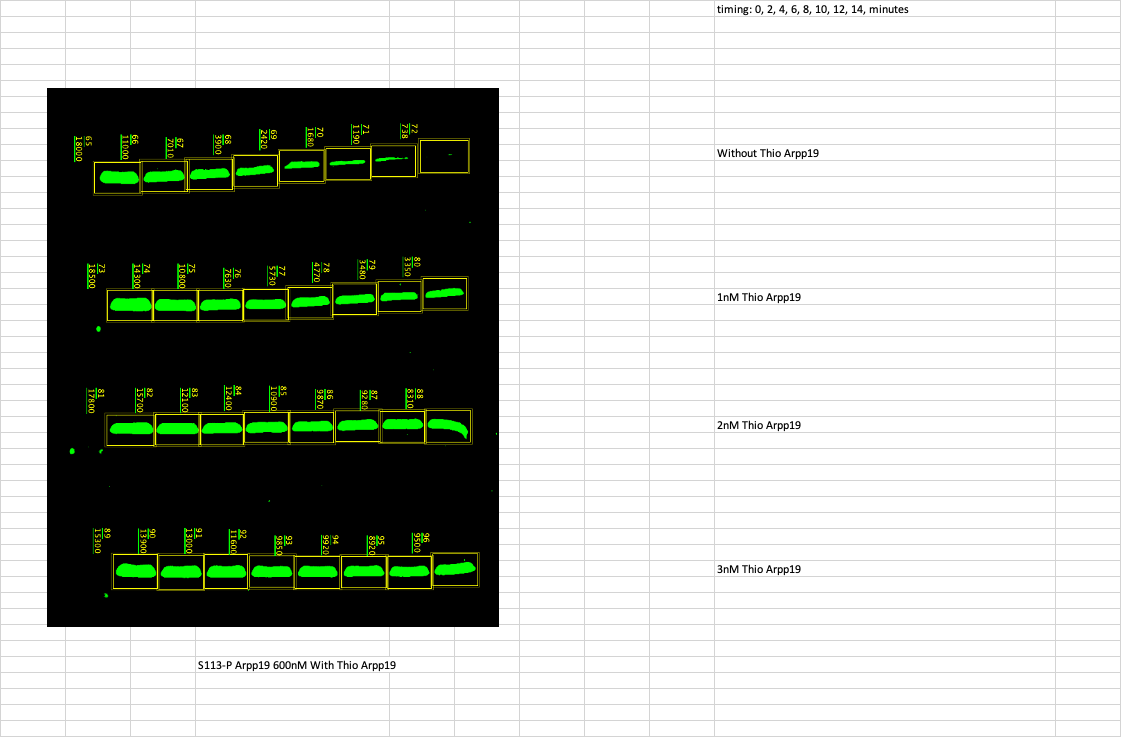

Supplement: Supplementary file 9 — Source Data Appendix Fig. S1_S6 [file 44318_2024_54_MOESM9_ESM.zip › Supp. Figure 3/supp. Figure 3a/western S113-P Arpp19 600nM + Thio Arpp19.tif]

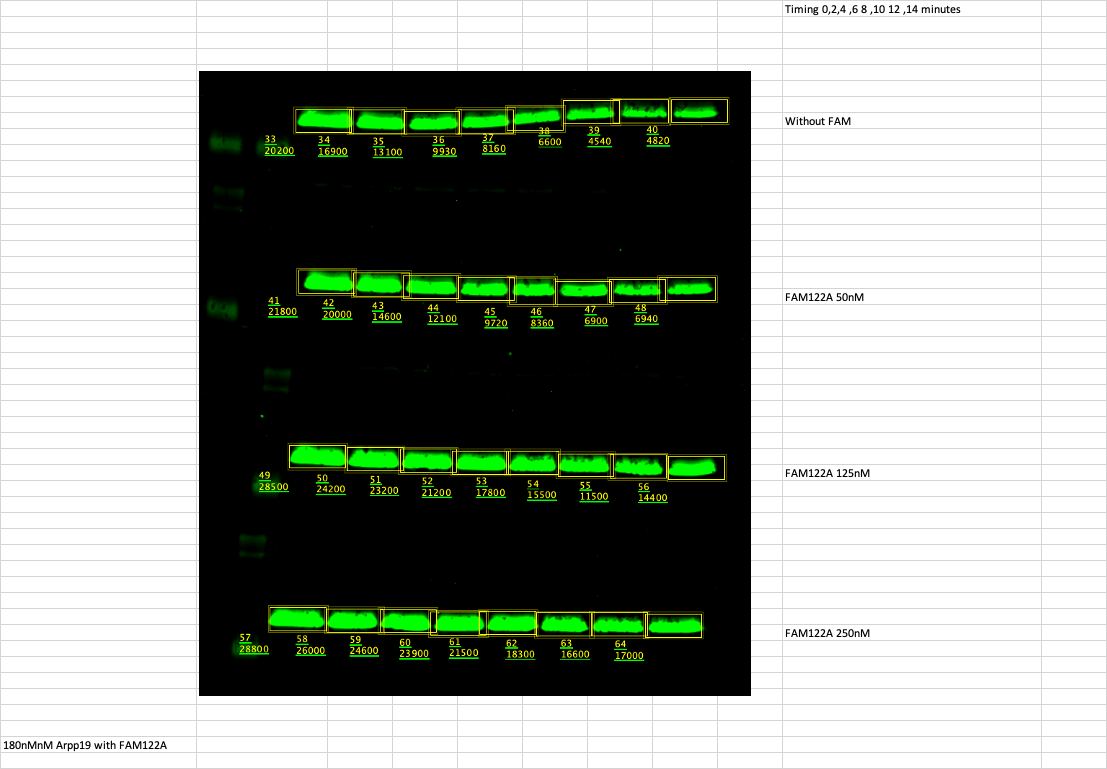

Supplement: Supplementary file 9 — Source Data Appendix Fig. S1_S6 [file 44318_2024_54_MOESM9_ESM.zip › Supp. Figure 3/supp. Figure 3a/western P113-Arpp19 180nM + FAM.tif]

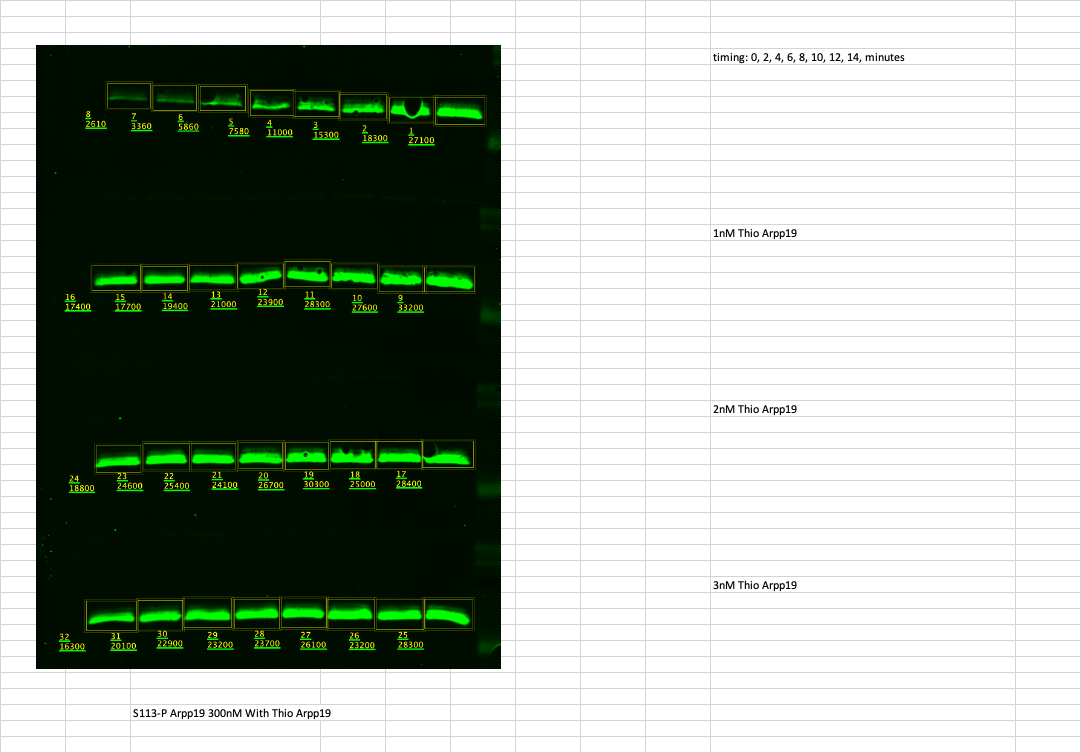

Supplement: Supplementary file 9 — Source Data Appendix Fig. S1_S6 [file 44318_2024_54_MOESM9_ESM.zip › Supp. Figure 3/supp. Figure 3a/Western S113-PArpp19 300nM +thio Arpp19.tif]

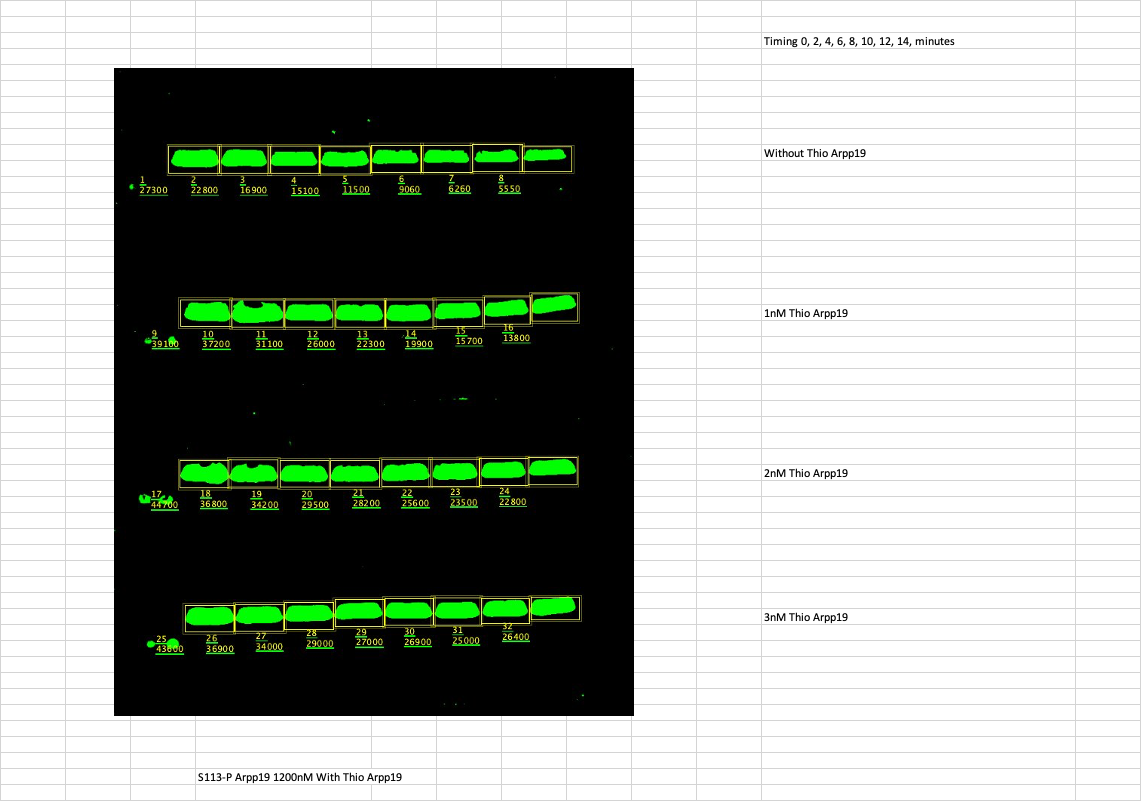

Supplement: Supplementary file 9 — Source Data Appendix Fig. S1_S6 [file 44318_2024_54_MOESM9_ESM.zip › Supp. Figure 3/supp. Figure 3a/western S113-PArpp19 1200nM + ThioArpp19.tif]

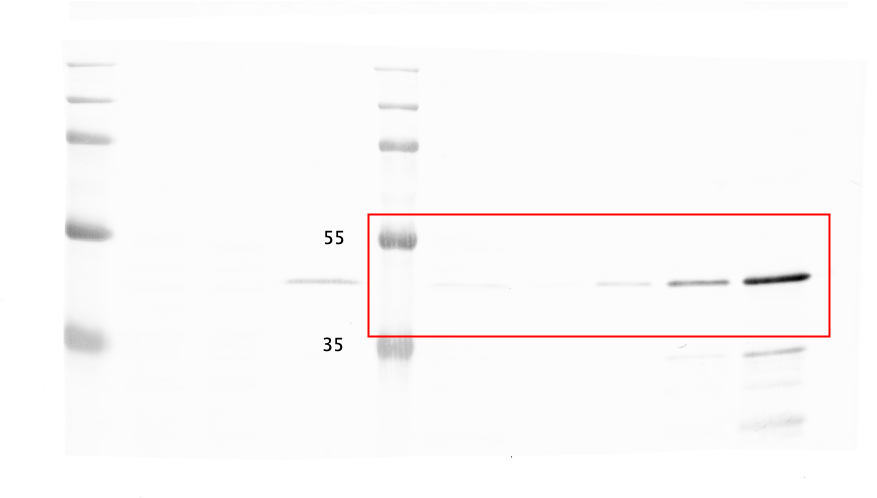

Supplement: Supplementary file 9 — Source Data Appendix Fig. S1_S6 [file 44318_2024_54_MOESM9_ESM.zip › Supp. Figure 5/supp. Figure 5a/FAM122A_quantif_cells_annotated.jpg]
