# Supplementary material for: Increases in cyclin A/Cdk activity and in PP2A-B55 inhibition by FAM122A are key mitosis-inducing events
Source: EMBO J. 2024 Feb 20;43(6):993–1014. doi: 10.1038/s44318-024-00054-z (PMC10943098; doi:10.1038/s44318-024-00054-z)
Supplement: Supplementary file 9 — Source Data Appendix Fig. S1_S6 [file 44318_2024_54_MOESM9_ESM.zip › Supp. Figure 1/Comments on supp.Figure 1.docx]

The samples taken as a function of time are those shown and depicted in Supp Figure 1 a and b.
