# Supplementary material for: Increases in cyclin A/Cdk activity and in PP2A-B55 inhibition by FAM122A are key mitosis-inducing events
Source: EMBO J. 2024 Feb 20;43(6):993–1014. doi: 10.1038/s44318-024-00054-z (PMC10943098; doi:10.1038/s44318-024-00054-z)
Supplement: Supplementary file 9 — Source Data Appendix Fig. S1_S6 [file 44318_2024_54_MOESM9_ESM.zip › Supp. Figure 3/Comments on supp. Figure 3.docx]

This data was generated to calculate the Km and Ki. Timepoints are taken every minute from 0 to 7 minutes for S113-P Arpp19 120nM and 180nM concentrations. For S113-P Arpp19 concentrations of 300nM, 600nM, 1200nM and 2000nM, time points are taken every 2 minutes.

Finally, time course 0 corresponds to the highest (intensity) value and Time point 7 or 14 corresponds to the lowest (intensity) value.
